# misFinder: identify mis-assemblies in an unbiased manner using reference and paired-end reads

**DOI:** 10.1186/s12859-015-0818-3

**Published:** 2015-11-16

**Authors:** Xiao Zhu, Henry C. M. Leung, Rongjie Wang, Francis Y. L. Chin, Siu Ming Yiu, Guangri Quan, Yajie Li, Rui Zhang, Qinghua Jiang, Bo Liu, Yucui Dong, Guohui Zhou, Yadong Wang

**Affiliations:** College of Computer Sciences and Information Engineering, Harbin Normal University, Harbin, Heilongjiang China; Center for Bioinformatics, School of Computer Sciences and Technology, Harbin Institute of Technology, Harbin, Heilongjiang China; Department of Computer Science, University of Hong Kong, Pokfulam Road, Hong Kong, China; The Fourth Affiliated Hospital of Harbin Medical University, Harbin, Heilongjiang China; School of Life Science and Technology, Harbin Institute of Technology, Harbin, Heilongjiang China; Department of Immunology, Harbin Medical University, Harbin, Heilongjiang China

## Abstract

**Background:**

Because of the short read length of high throughput sequencing data, assembly errors are introduced in genome assembly, which may have adverse impact to the downstream data analysis. Several tools have been developed to eliminate these errors by either 1) comparing the assembled sequences with some similar reference genome, or 2) analyzing paired-end reads aligned to the assembled sequences and determining inconsistent features alone mis-assembled sequences. However, the former approach cannot distinguish real structural variations between the target genome and the reference genome while the latter approach could have many false positive detections (correctly assembled sequence being considered as mis-assembled sequence).

**Results:**

We present misFinder, a tool that aims to identify the assembly errors with high accuracy in an unbiased way and correct these errors at their mis-assembled positions to improve the assembly accuracy for downstream analysis. It combines the information of reference (or close related reference) genome and aligned paired-end reads to the assembled sequence. Assembly errors and correct assemblies corresponding to structural variations can be detected by comparing the genome reference and assembled sequence. Different types of assembly errors can then be distinguished from the mis-assembled sequence by analyzing the aligned paired-end reads using multiple features derived from coverage and consistence of insert distance to obtain high confident error calls.

**Conclusions:**

We tested the performance of misFinder on both simulated and real paired-end reads data, and misFinder gave accurate error calls with only very few miscalls. And, we further compared misFinder with QUAST and REAPR. misFinder outperformed QUAST and REAPR by 1) identified more true positive mis-assemblies with very few false positives and false negatives, and 2) distinguished the correct assemblies corresponding to structural variations from mis-assembled sequence. misFinder can be freely downloaded from https://github.com/hitbio/misFinder.

**Electronic supplementary material:**

The online version of this article (doi:10.1186/s12859-015-0818-3) contains supplementary material, which is available to authorized users.

## Background

The high throughput sequencing (HTS) technologies [1, 2] have been a major transformation in the way scientists extract genetic information from biological systems, revealing limitless insight about the genome, transcriptome, and epigenome of many species. One major step of analysis is combining the overlapped reads (fragments of DNA sampled from genomes) to reconstruct the original genome sequence of the target species, called *assembly*. However, the lengths of reads (typically 50–250 base pairs [3, 4]) generated by HTS technologies are much shorter than those of the traditional Sanger sequencing (typically about 800 base pairs [5]) and the sequencing error rate is usually higher (1-2 % compared with 0.1 %). In addition, there are many repetitive sequences along the genomes. All the above features will make the assembling process difficult and will introduce mis-assembled genome sequence.

Many assembly methods [6–12] have been developed to deal with these challenges and they have steadily improved in recent years. However, the problem of mis-assembly is still unsolved [13] and the assembly errors will have adverse impact to downstream analysis [14]. There are two main approaches for determining mis-assembled sequences depending on whether we have a similar reference genome for the target genome (the genome we perform sequencing).

### Reference-based approach

If the genome reference for the target organism is available, mis-assembled sequences, e.g., misjoins and erroneous insertions/deletions, can be detected by comparative analysis of the reference sequence and the assembled genome sequence. However, a good genome reference is usually not available. When the target genome and the reference genome are not the same, there are some differences caused by structural variations between them. Mauve [15] regards these differences as assembly errors directly without further analysis, which may affect the final result (many false positive mis-assembly detections). GAGE [14] and QUAST [16] regard these differences as erroneous indels (i.e., erroneous insertions/deletions) or misjoins. All these tools do not distinguish whether the differences caused by assembly errors or by structural variations, therefore further analysis is required to determine which differences are due to mis-assembly to prevent introducing false error calls.

### *De novo* approach

If a reference is unavailable, the alignment of the raw reads to their assembly provides indirect measures of assembly quality, e.g., high variation in coverage depth alone mis-assembled sequence, inconsistent insert distance when aligning paired-end reads to mis-assembled sequence, etc. This information can then be used to detect single-base changes, repeat condensation or expansion, false segmental duplications and other mis-assemblies. CGAL [17] and ALE [18] both produce a summary likelihood score of an assembly. ALE also reports four likelihood scores for each base representing the probability that an assembly is correct. However, they lack the ability to transform metrics to accurate error calls [19]. REAPR [19] reports a single score for each base for accuracy derived from just a few metrics, such as the coverage depth distribution, it may tend to introduce some false error calls since the real reads data usually are uneven, and it also tends to break the scaffolds in their gap regions by mistake (false errors).

Both reference-based and *de novo* approach may partially solve the mis-assembly problem. However, reference-based approach cannot distinguish real structure variations and mis-assembled sequences while *de novo* approach can have many false positive detections because of the uneven sampling in real data. A better performance can be achieved by combining both approaches in determining mis-assembled sequences.

In this article, we present misFinder, a tool that aims to identify the assembly errors with high accuracy in an unbiased way (unbiased means that distinguish the assembly errors and correct assemblies corresponding to structural variations, without introducing false error detections) and correct these errors at their mis-assembled positions to improve the assembly accuracy for downstream analysis. It uses the reference (or close related reference) to find the differences between the scaffolds and the reference, and uses paired-end reads to validate these differences to determine whether they are assembly errors or correct assemblies corresponding to structural variations rather than regarding them as errors directly with some biases. In order to distinguish the assembly errors and correct assemblies corresponding to structural variations, misFinder analyzes the patterns of each type of assembly errors, and then applies multiple features derived from coverage and consistence of paired-end reads for these errors to obtain high confident error calls and pinpoint the correct assemblies corresponding to structural variations, thus resulting assembly error calls with high accuracy in an unbiased way.

## Methods

**Fig. 1:**
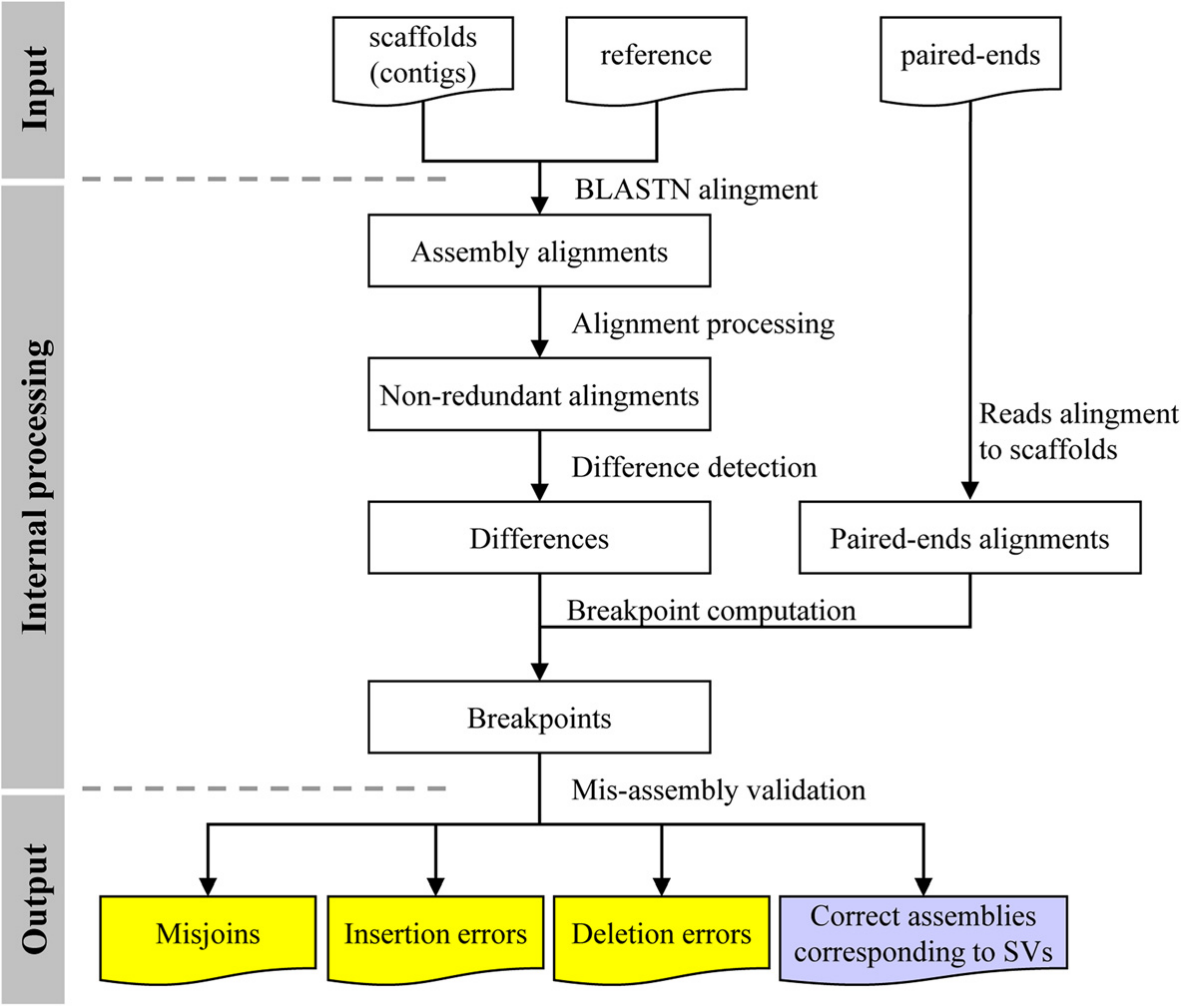
Workflow of misFinder. MisFinder consists of three major steps. (1) Identify putative mis-assembles. Scaffolds (contigs) and genome reference are first used to generate the BLASTN alignments followed by the alignment processing that the redundant alignments will be removed, and then the putative mis-assembles are identified according to their non-redundant alignments. (2) Breakpoint computation. Paired-end reads are aligned to the scaffolds to make the breakpoints more accurate. (3) Mis-assembly validation. Putative mis-assemblies are validated according to the alignment information of paired-end reads on scaffolds

The workflow of misFinder to identify assembly errors is shown in Fig. 1. misFinder is based on BLASTN [20], assembly (contigs/scaffolds), genome reference and paired-end reads from Solexa sequencing technology are its input, with aims to identify the mis-assemblies i.e., the assembly errors, and correct these errors to increase the accuracy of the assembly. It consists of three major steps: (1) Identify the differences between scaffolds and reference using BLASTN alignment; (2) Compute the breakpoints according to paired-end reads alignment information; (3) Validate the differences according to paired-end reads alignment information to distinguish the assembly errors and correct assemblies corresponding to structural variations. The algorithm of misFinder will be described in details in the following sections.

### Identify differences between scaffolds and reference

#### Align scaffolds to reference

misFinder is based on the alignments of BLASTN (version 2.2.25+ and higher) [20], a well-established long-range nucleotide sequence alignment program for producing accurate long-range sequence alignments, which is particularly suitable for capturing the scaffolds alignment information. As BLAT [21] does not produce single hit for distances >750 kbp [22], and according to our experiments, it tends to break single well-aligned segments into multiple small pieces as it produces gapped alignments, and the most important, it may miss well-aligned segments sometimes, while these situations does not occur for BLASTN which is well-known and always tries to produce continuous alignments as large as possible. Therefore, we choose BLASTN as our long-sequence alignment tool.

misFinder uses BLASTN in multiple threads approach to compute the alignments, First, the scaffolds are divided into equal-sized parts to run BLASTN independently with multiple threads; and then, the sub-alignments for each thread are merged to generate the whole alignment results for further analysis.

#### Obtain non-redundant alignments

If a scaffold is aligned to genome reference uniquely, we keep its alignment information without additional requirements. Otherwise, we select the several best aligned segments that cover the whole scaffold, and other alignments are removed as they are short and redundant to the alignment of the whole scaffold. As the alignments from BLASTN are already sorted in descending order according to their scores, so we first choose the best aligned segment as the start segment, and select its adjacent segments one by one to the 3′ end of the scaffold until no adjacent ones, and then the adjacent ones are selected to the 5′ end of the scaffold in the same way. For selecting the adjacent segment, the candidates should have an overlap or a gap no more than 1 kbp by default according to the BLASTN alignments, and select the one of the minimal overlap or gap size. If there are no adjacent segments, the segment that has the minimal overlap or gap size according to the scaffold position is selected. These selected segments cover the whole scaffold and are placed according to their positions in scaffold. Other unselected segments are redundant and are removed.

#### Identify differences using BLASTN

**Fig. 2:**
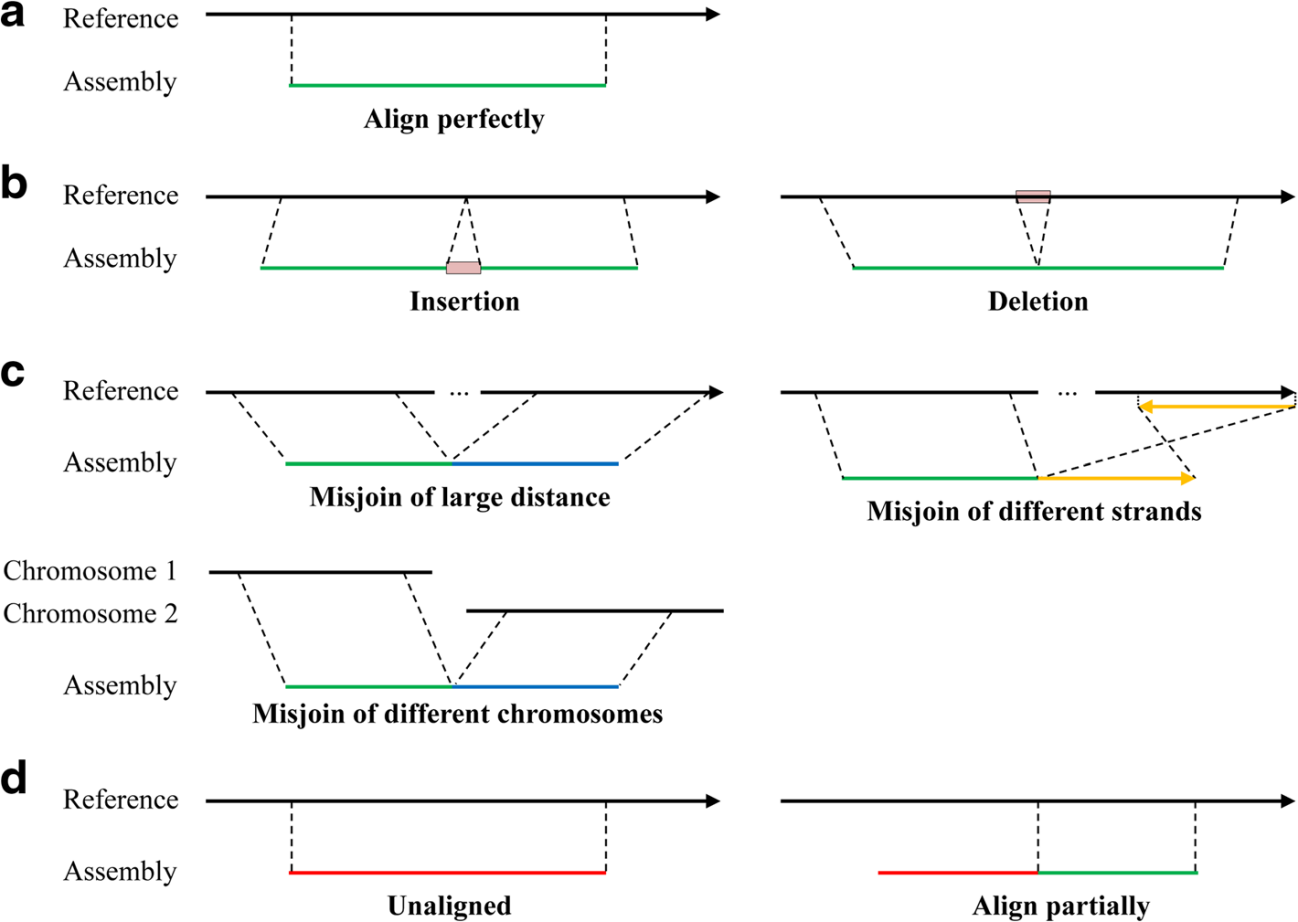
Overview of differences between scaffolds and reference according to BLASTN alignments. Solid lines indicate reference (top) and scaffolds (bottom) respectively, red lines indicate unaligned segments, dashed lines between reference and scaffolds indicate alignment borders. **a** Scaffold is perfectly aligned to the genome reference. **b** Insertion or deletion error in scaffolds. An insertion/deletion error in scaffold causes alignment breaks, and the scaffold will be split into several aligned segments separated by the insertion/deletion error. **c** Misjoin in scaffold. Assembly errors caused by joining distinct genome regions with large distance (top left), by joining segments which can be aligned to different reference strands (top right), or by joining segments between different chromosomes (in eukaryotes) or between chromosome and plasmid (in bacteria) (bottom left). The yellow solid line indicates the region of reverse strand of the reference region. **d** Scaffolds that have entirely no alignment information (left) or have partial unaligned segments relative to the scaffold (right). We treat these situations as insertions that may be caused by assembly errors or by novel sequences in target genome

After analyzing the non-redundant alignment information of scaffolds generated by BLASTN, four kinds of patters are observed (Fig. 2). Some scaffolds can perfectly match with reference (Fig. 2a), and there will be no assembly errors within these scaffolds. Other three most common kinds of differences, i.e., misjoin, insertion and deletion, may be due to assembly errors (Fig. 2b-d). The misjoin may be caused by joining segments with large distance or different strands in genome, or by joining segments between different chromosomes (e.g., in eukaryotes) or between chromosome and plasmid (in bacteria). The insertion/deletion may be caused by incorrectly expanded/collapsed repetitive sequences during assembly, or may be caused by structural variation between the target genome and reference genome. Note that the scaffold segments with no alignment information are treated as insertion, and they may be caused by sequencing errors or by novel sequences compared to the genome reference. BLASTN tends to break the alignment at these regions of differences, and these differences have high possibility to be assembly errors. misFinder identifies these differences as putative mis-assemblies which needs further validations to determine whether they are true assembly errors.

### Compute breakpoints

After analyzing the alignments of the scaffolds to the genome reference, three common types of mis-assemblies, including misjoin, erroneous insertion and erroneous deletion, are observed at the positions of differences according to their alignment information. Before calling assembly errors, it is necessary to get the scaffold regions which have differences that may contain assembly errors for misjoin, erroneous insertion/deletion. We call such scaffold region as *breakpoint region* with left margin *M*_*L*_ and right margin *M*_*R*_. According to our observations, the normal regions have well aligned read pairs and relative even read coverage depth (Additional file 1: Figure S1), and whereas for the three types of mis-assemblies, they have different error patterns in their breakpoint regions, which can be used for error calling (see section “Validate assembly errors”).

Since the target genome and the reference genome are not exactly the same, there are usually some differences between them, i.e., structural variations (SVs). Therefore, in order to distinguish assembly errors and correct assemblies corresponding to structural variations, paired-end reads are aligned to scaffolds to call the mis-assemblies using multiple kinds of information extracted from the paired reads data (see section “Validate assembly errors”). Note that, for the reads fall into repeat regions, their best alignments are randomly selected to prevent the correct regions from having zero read coverage, and we call these reads with multiple aligned positions as *multiple aligned reads*, misFinder marks these multiple aligned reads for further analysis.

#### Breakpoint region for misjoins

**Fig. 3:**
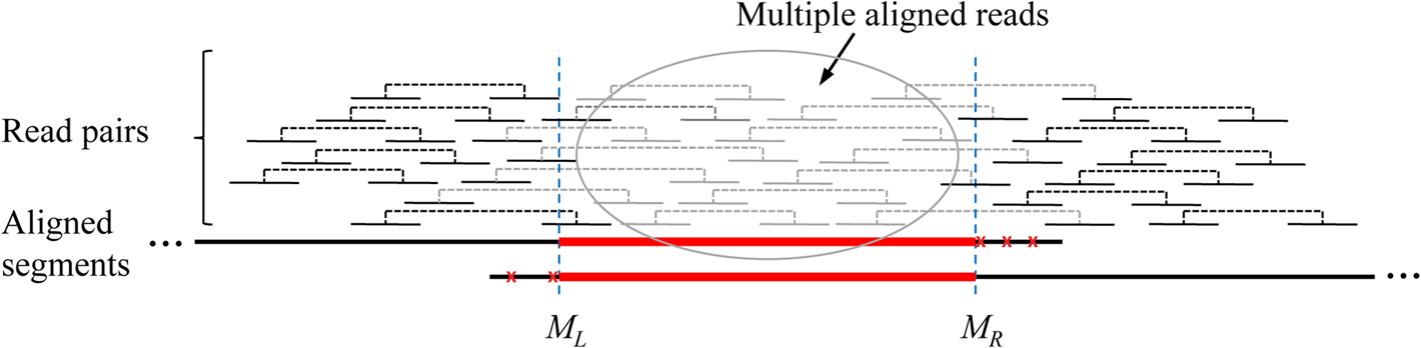
Misjoin in scaffold. After aligning the misjoined scaffold to reference, it will be split into two sub-segments according to the alignment information, and the two sub-segments are aligned with different reference strands or aligned to distinct regions with long distance. And these two sub-segments overlap each other due to the repeat (middle thick red lines) whose erroneous tails around margins *M*_*L*_ and *M*_*R*_ will be trimmed

Misjoin in scaffold can be depicted in Fig. 3. For the scaffold with misjoin, the whole scaffold will be divided into several large segments after BLASTN alignment, and these segments can be aligned to different reference regions respectively, with different strands or long distance of distinct regions, e.g., more than several kilo bases, or can be aligned to different chromosomes (e.g., in eukaryotes) or between chromosome and plasmid (in bacteria). The misjoins are caused by repeats which are not correctly resolved by assembly algorithm.

As BLASTN usually produces some mismatched bases around repeat margins *M*_*L*_ and *M*_*R*_, misFinder trims the segment tails containing mismatched bases to get the margins *M*_*L*_ and *M*_*R*_ (Fig. 3). This repeat region between *M*_*L*_ and *M*_*R*_ is a breakpoint region that may contain assembly errors. For BLASTN alignments, the adjacent two misjoined segments usually have some overlaps, and it is observed that there are many multiple aligned reads (reads with multiple aligned positions in scaffolds) in the region, and the scaffold should be split into two small pieces if the breakpoint region contains a misjoin assembly error.

#### Breakpoint region for erroneous insertions

**Fig. 4:**
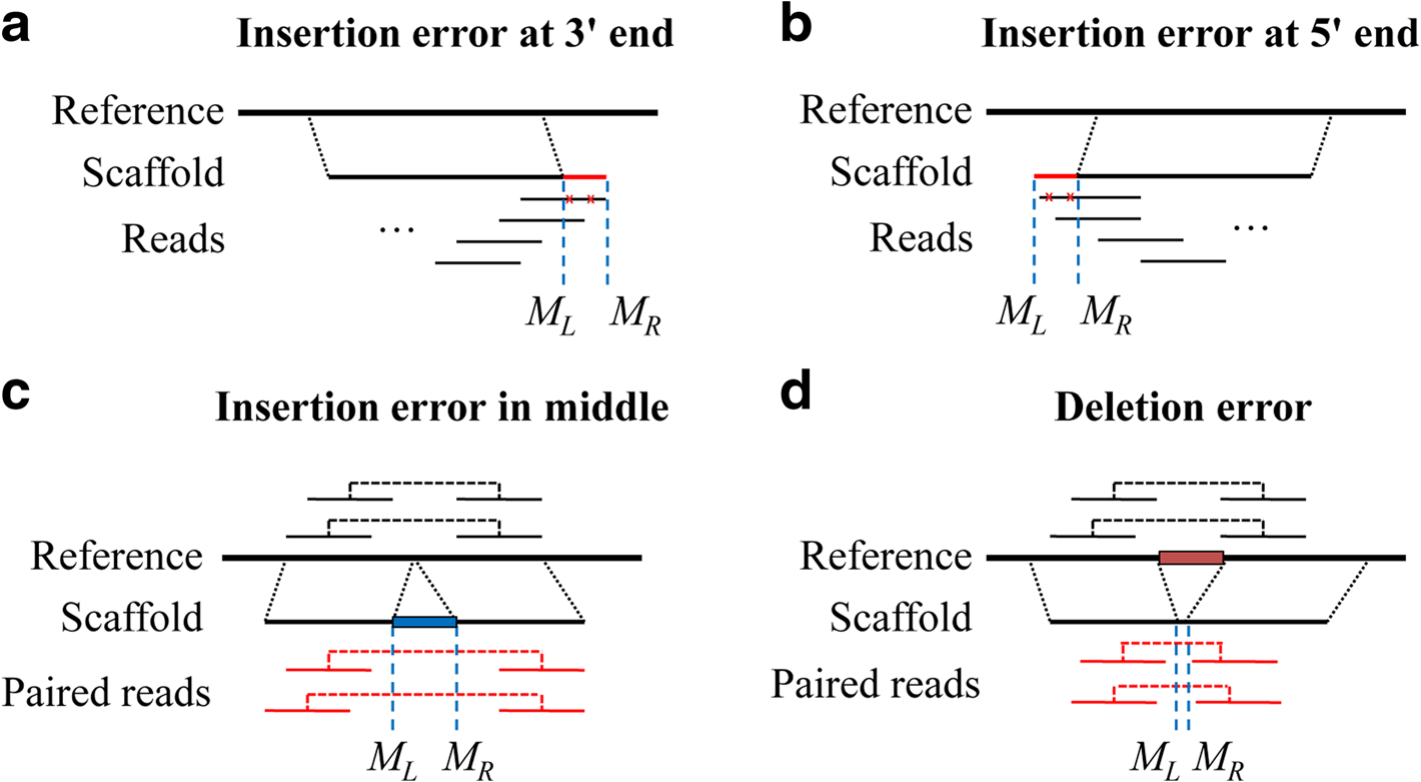
Insertion/deletion error in scaffold. **a**-**b** An insertion error at scaffold end. There are usually only single-end reads can be aligned to the erroneous ends. **c** An insertion error in the middle of two well-aligned segments whose paired-end reads have a larger insert size than normal regions. **d** A deletion error in the middle of two well-aligned segments whose paired-end reads have a smaller fragment size than normal regions. *M*_*L*_ and *M*_*R*_ are the left margin and the right margin for the insertion (or deletion) error, respectively

Scaffolds usually have some erroneous insertions in the middle, and they also usually have some unaligned segments at the ends, we treat all of the above two cases as insertion errors, which can be illustrated in Fig. 4a-c. For the insertion error at the end of scaffolds, the end usually contains some erroneous bases, and there are usually only single-end reads covering the breakpoint region without paired-end reads. For the insertion error in the middle of scaffold, it has two flanking well-aligned segments at both sides of it, and the paired-end reads aligned on the two segments have a larger insert size than normal regions. The insertion error has a breakpoint region with margins *M*_*L*_ and *M*_*R*_ which can be easily determined from alignments directly.

#### Breakpoint region for deletions

Scaffolds also usually contain some erroneous deletions which can be illustrated in Fig. 4d. Unlike erroneous insertion, the erroneous deletion is a missing sequence with two well-aligned segments around it, and the aligned paired-end reads on the two segments have a shorter insert size than that of normal regions. The deletion error also has a breakpoint region with margins *M*_*L*_ and *M*_*R*_ which can be easily determined from alignments directly.

### Validate assembly errors

After aligning paired-end reads to scaffolds, there are several features that are different with the normal regions for these putative mis-assemblies, including abnormal (high or low) coverage, some disagreements (majority nucleotide differs no much with others), multiple aligned reads (reads with multiple aligned positions), fragment size difference (difference of distance of two 5′ ends of a read pair and the library insert size), and discordant read pairs (erroneous orientations or abnormal fragment size difference, e.g., >3 * standard deviation). The mis-assembled regions typically have high or low coverage (even zero coverage), much more discordant read pairs than other normal regions. So that, the putative mis-assemblies could be validated by using the paired-end reads aligned to the scaffolds. The *high coverage* means that the breakpoint region has more than one 50-bp sub-region whose coverage is higher than 1.5 fold of the average coverage of that scaffold, while the *low coverage* means that it has more than one 50-bp sub-region whose coverage is lower than 0.5 fold of the average coverage of that scaffold. And for the discordant read pairs, we calculate the ratio of the discordant read pairs and all the read pairs in the breakpoint region, named *discordant ratio*, and the breakpoint region with much more discordant read pairs usually has a high discordant ratio, say >0.1. And for the breakpoint region larger than 500 base pairs, we calculate the discordant ratio for each 500-bp sub-region.

As the single-cell data typically have highly uneven sequencing depth, we re-define the sub-regions of high coverage and low coverage in the case of single-cell sequencing data. For a scaffold, we compute the average coverage of each 50-bp sub-region, and then calculate the mean coverage and standard deviation of the coverage of these sub-regions of the scaffold. The *high coverage* means that the breakpoint region has more than one 50-bp sub-region whose coverage is at least 1.5 fold of the standard deviation higher than the mean coverage of that scaffold, while the *low coverage* means that it has more than one 50-bp sub-regions whose coverage is at least 1.5 fold of the standard deviation lower than the mean coverage of that scaffold.

Since different errors appear different patterns, we applied different methods to validate their correctness. The misjoins could be validated using the abnormal coverage, disagreements, multiple aligned reads, and the insertion/deletion errors could be validated using the abnormal coverage, discordant read pairs and the fragment size difference.

#### Validate misjoins

After aligning paired-end reads to scaffolds, it is observed that there are some differences between the breakpoint region (repeat region) and other normal regions around the margins *M*_*L*_ and *M*_*R*_ (Fig. 3, Additional file 1: Figures S2-S3). The patterns of misjoins are described as below.

In misjoin regions, there are usually some disagreements and abnormal coverage around margins *M*_*L*_ and *M*_*R*_. This is caused by the repeats that come from different genome regions which are incorrectly joined by assembly algorithm. Even though one end of the paired-end reads can be aligned to the scaffold, the other end may be aligned with some mismatched bases which usually have the same position in scaffold (Fig. 3, Additional file 1: Figures S2-S3). For a base in scaffold, there will be many aligned reads covering it, the read count is called *coverage* which consists of the counts of A, C, G and T, respectively. If the majority nucleotide differs no much with others, e.g., the percent of the majority <0.8, we call the nucleotide in scaffold a *disagreement*. For misjoins, there are usually some disagreements around breakpoint margins *M*_*L*_ and *M*_*R*_, whereas the normal regions usually have no disagreements (Additional file 1: Figure S1). Also, for the breakpoint region with margins *M*_*L*_ and *M*_*R*_, some nucleotides may also have abnormal high or low coverage (even zero coverage), and these abnormal coverage usually indicates assembly error.

After aligning paired-end reads to scaffolds, most of the reads are uniquely aligned in normal regions, and there are usually some multiple aligned reads in misjoin regions with margins *M*_*L*_ and *M*_*R*_ (Fig. 3). This is also caused by repeats, the repeat regions usually have more multiple aligned reads than that of other normal regions. Therefore, for a breakpoint region of misjoin, we compute the percentage of the multiple aligned reads, named *multi-align ratio*, to determine whether this difference is caused by repeats. High values of the multi-align ratio (e.g., >0.1) may indicate the assembly error.

Therefore, in summary, the putative misjoin is a validated assembly error if it satisfies at least one of the following two conditions: (1) it has abnormal coverage or more than 1 disagreement; (2) the multi-align ratio is high, say >0.1. Otherwise, it may be due to structural variation that needs further analysis (see section “Distinguish correct assemblies corresponding to structural variations”).

#### Validate insertion errors

After placing paired-end reads to their most likely locations in scaffolds and computing the breakpoint regions of insertion errors (Fig. 4a-c), misFinder analyzes the aligned reads around the breakpoint regions. For the insertion error in the middle, misFinder identifies the paired-end reads whose two ends are aligned to the two flanking sub-segments, and then calculates the fragment size of the paired-end reads. If the difference of the fragment size and the library insert size is close to the size of the inserted sequence, e.g., <2 * standard deviation, then it may indicate an assembly error. Moreover, it may contain some disagreements, low coverage, and many discordant read pairs with large fragment size (Additional file 1: Figure S4). Therefore, in order to call the assembly error with high confidence, the putative insertion error in the middle of a scaffold is a validated assembly error if: (1) the difference of the fragment size and the library insert size is close to the size of the inserted sequence, and (2) it satisfies at least two of the following conditions: (i) it has disagreements; (ii) low coverage; and (iii) many discordant read pairs with large fragment size, e.g., discordant ratio > 0.1.

For the insertion errors whose differences of the fragment size and the library insert size are not close to the size of the inserted sequences, the length of inserted sequences are usually larger than the read length. They usually have some disagreements in the breakpoint regions, and we calculate the number of disagreements per kilo base pairs in the breakpoint regions. Therefore, these putative insertion errors in the middle of scaffolds are validated assembly errors if they have more than one disagreement per kilo base pairs in their breakpoint regions.

Moreover, for the insertion error at the 5′/3′ end of a scaffold, as it has no paired-end reads for most cases, misFinder calculates the read coverage at the scaffold end, and if there are some disagreements or zero-coverage nucleotides, then it should be a true erroneous insertion sequence.

#### Validate deletion errors

In contrast with insertion error, the deletion error has a missing sequence in scaffolds (Fig. 4d). misFinder identifies the paired-end reads whose two ends are aligned to the two flanking sub-segments, and then calculates the fragment size of these paired-end reads. If the difference of the library insert size and the fragment size is close to the size of the deleted sequence, e.g., <2 * standard deviation, then it may indicate an assembly error. It may contain some disagreements, high or low coverage (even zero-coverage), and some discordant read pairs with erroneous orientation or small fragment size in the breakpoint region (Additional file 1: Figure S5). Therefore, in order to call the assembly error with high confidence, the putative deletion error is a validated assembly error if: (1) the difference of the library insert size and the fragment size is close to the size of the deleted sequence, and (2) it satisfies at least two of the following conditions: (i) it has disagreements; (ii) high or low coverage (even zero-coverage); and (iii) many discordant read pairs with erroneous orientation or small fragment size, e.g., discordant ratio > 0.1.

Similar with the insertion errors, for the deletion errors whose differences of the fragment size and the library insert size are not close to the size of the deleted sequences, the length of deleted sequences are usually larger than the read length. They usually have some disagreements in the breakpoint regions, and we calculate the number of disagreements per kilo base pairs in the breakpoint regions. Therefore, these putative deletion errors in the scaffolds are validated assembly errors if they have more than one disagreement per kilo base pairs in their breakpoint regions.

### Distinguish correct assemblies corresponding to structural variations

After validating the potential mis-assemblies, assembly errors are identified, and there are still some differences due to structural variations that are not validated as assembly errors, and they may need further analysis. In order to distinguish these correct assemblies corresponding to structural variations with high confidence, we perform the correct assembly analysis to determine them.

For the correctly assembled scaffold regions, they usually have the same patterns as the normal regions, while for assembly errors their patterns differ significantly with the normal regions, which can be used to determine the correct assemblies from the differences that are not been identified as assembly errors in previous section. For the difference between the scaffold and reference that is not identified as assembly error, if its breakpoint region has even coverage, no disagreements, and very few discordant read pairs (e.g., discordant ratio <0.1), it should be a correct assembly whose difference is caused by structural variations; otherwise, misFinder output it as a warning that may need further analysis.

After the analysis, some novel sequences could be identified, e.g., novel sequences of 9 kbp and 4.6 kbp in *S.pombe* strain jb1168 were identified, and they should be correct assemblies rather than assembly errors (Additional file 1: Figures S6-S7).

## Results

### Detection of assembly errors on simulated data

Genome assembly usually contains some common errors such as misjoins of distinct genomic regions, erroneous insertions and deletions. To test misFinder’s ability to detect such errors, we used GemSIM [23] to generate 50× simulated Illumina short reads data on *Escherichia coli* K12 MG1655 (refSeq: NC_000913.2, genome size 4.64 Mbp), with mean insert size 368 bp and standard deviation 61 bp. Next, these paired-end reads data were assembled using MaSuRCA (v2.2.1) [24], and 71 scaffolds were generated. Finally, the misFinder was performed to identify assembly errors in the scaffolds using the genome reference and paired-end short reads data. Note that the differences between the assembly and reference could be directly treated as errors without considering structural variations for simulated reads data.

According to our experiments, MaSuRCA produced more assembly errors (especially the fatal misjoins) than other assemblers on *E.coli* and *S.pombe* genomes. MaSuRCA generated more mis-assembled contigs than other assemblers, and the detailed information can be seen in our previous work [12], therefore, MaSuRCA was chosen to be the assembler for the experiments of *E.coli* and *S.pombe* genomes to give better presentations for the performance of misFinder on identifying the assembly errors and structural variations.

**Fig. 5:**
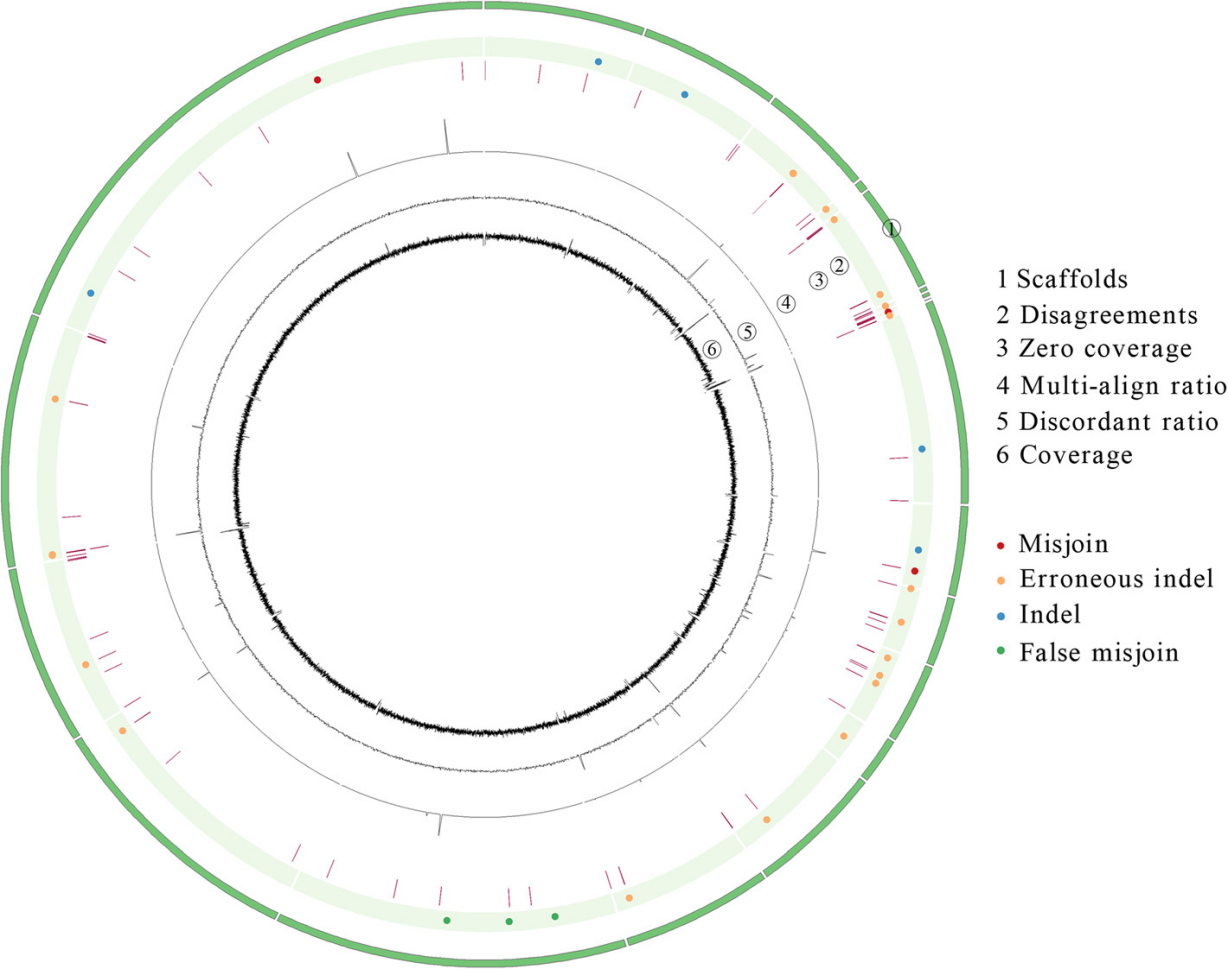
Visualization of misFinder output for identifying assembly errors on *E.coli* simulated data. The running results of misFinder are shown using Circos [30]. The ideogram (green) shows the circularized selected scaffolds containing errors and structural variations. The scatter plot shows the identified assembly errors (red circles for misjoins, orange circles for indel errors) and correct assemblies (blue circles for correct indels, green circles for false misjoins) corresponding to structural variations by misFinder. There are 27 assembly errors and 8 correct assemblies corresponding to artificial SVs. The disagreement plot marks the disagreement for each base in scaffolds. The zero coverage plot marks each nucleotide with zero coverage. The multi-align ratio plot shows the ratio of multiple aligned reads for each region of 500 bp, ranging from 0 to 1. The discordant ratio plot shows the discordant ratio of discordant read pairs for each 500-bp region in scaffolds, ranging from 0 to 1. The last plot shows the read coverage in scaffolds

We tested misFinder by first aligning the assembly to the reference using BLASTN (v2.2.25+) [20] with option ‘-best_hit_overhang 0.1’ to reduce the redundant short align segments. The remaining redundant short segments were further removed to obtain non-redundant align segments, therefore most of the scaffolds each had only one large align segment with only a few mismatched bases, e.g., insertions/deletions and mismatches. Then, the paired-end reads were aligned to the assembly to assist in extracting the suspicious mis-assembly breakpoint regions before analyzing their alignment information. Finally, 27 assembly errors including 3 misjoins, 20 insertion errors and 4 deletion errors were identified according to multiple features of their paired-end reads information (Fig. 5).

We checked all these identified assembly errors manually, and found all of them were true assembly errors and correctly identified by misFinder without introducing mis-identification errors, which may indicate misFinder’s high accuracy of identification of assembly errors.

### Distinguish assembly errors and correct assemblies corresponding to structural variations on simulated data

Since the target genome and the reference genome usually are not the same, they may contain some structural variations (SVs), and for the difference between the assembly and reference, it may be caused by assembly errors or it is a correct assembly corresponding to SVs, therefore it needs to determine whether the difference is caused by assembly error or it is a correct assembly corresponding to SVs when performing the assembly error call.

To test the ability of misFinder to call assembly errors and correct assemblies corresponding to SVs, we introduced six different modifications into the *E.coli* genome reference to analog the SVs, these modifications included one duplicated sequence (segment size 1 kbp), one large relocation (segment size 57 kbp), two insertions (70 bp and 30 bp) and two deletions (70 bp and 30 bp) (Additional file 1: Figure S8). The similarity between the artificially modified reference and the original reference is 99.97 %. We treated this mutated reference as new high quality reference, and treated the assembly as the target genome in which some differences may be due to SVs rather than assembly errors. Then, the scaffolds and paired-end reads data in previous section were directly used again to test the performance of misFinder on identification of the assembly errors and the correct assemblies with SVs that were correctly assembled. As the duplicated sequence introduced one difference and the large relocation produced three differences at their joined positions, there were eight differences between the target genome and the reference. As a result, misFinder successfully identified all the 27 assembly errors that were already detected in previous section without introducing miscalled errors, and moreover, it determined all the eight differences caused by SVs as correct assemblies (Fig. 5).

From the above two experiments on simulated short reads data, it shows that misFinder has good ability for identifying the assembly errors and correct assemblies corresponding to SVs with high accuracy by using the reference and paired-end reads data.

### Performance on *E.coli* single-cell data

We tested the performance of misFinder on *E.coli* K12 strain MG1655 single cell data. The single-cell sequencing data (*E. coli*, first single cell MDA, lane 1) [25] were downloaded from http://bix.ucsd.edu/projects/singlecell/nbt_data.html, with mean insert size 282 bp and standard deviation 65 bp, sequencing depth ~600×. The MaSuRCA assembly and the artificially modified reference in previous section were used again to test the performance of misFinder on identifying assembly errors and structural variations on highly uneven sequencing data.

The experiments for single-cell reads data were carried out on an Intel(R) Xeon(R) Core-16 CPU 2.67-GHz server supplied with 24 GB memory. The single-cell sequencing data option “-sc 1” was specified then performing the experiments using 16 threads, the running time was 8 min and the memory consumption was 2.4 GB, and all the 27 assembly errors and 8 structural variations were successfully identified without introducing other miscalls (result was not shown), which may indicate that misFinder has good performance not only on uniform distribution coverage data but also on highly uneven sequencing data (e.g., single-cell sequencing).

### Detection of assembly errors on real data

To test the performance of misFinder on real sequence data, we applied it to *Schizosaccharomyces pobme* strain jb1168 real short paired-end reads data by using the same assembler MaSuRCA [24]. The paired-end reads data (SRA: ERX174934) of *S.pombe* were downloaded from NCBI, with mean insert size 380 bp and standard deviation 82 bp. Since the reference of strain jb1168 is not available, we used the high quality reference of its close strain 972 h- (genome size 12.59 Mbp) which consists of three chromosomes and one mitochondrion (refSeqs: NC_003424.3, NC_003423.3, NC_003421.2 and NC_001326.1). For the assembly of the *S.pombe* strain jb1168, there were some assembly errors introduced during the assembly step, and moreover, the target genome and the reference genome were not exactly the same, there were some structural variations between them. Therefore, misFinder was used to find the assembly errors and correct assemblies corresponding to structural variations.

First, *de novo* assembly was carried out using MaSuRCA, and produced 465 scaffolds. Then, misFinder was applied to identify the assembly errors and distinguish the correct assemblies corresponding to SVs in the scaffolds by using the *S.pombe* strain 972h- reference and paired-end reads data. misFinder identified 116 assembly errors and 22 correct assemblies corresponding to structural variations with only three false positives and one false negative. For the 116 assembly errors, there were 22 misjoins, 49 erroneous insertions and 45 erroneous deletions; and for the 22 correct assemblies, there were 13 insertions, 6 deletions and 3 false misjoins, ranging from several base pairs to several kilos of nucleotides.

These assembly errors and correct assemblies corresponding to SVs were all manually checked by aligning their paired-end reads to see the coverage, disagreements and discordant read pairs and etc., using BLASTN [20] alignments and IGV [26]. For the 116 identified errors, there were only 3 false positives; and for the 22 correct assemblies, there was only one miscalls.

**Fig. 6:**
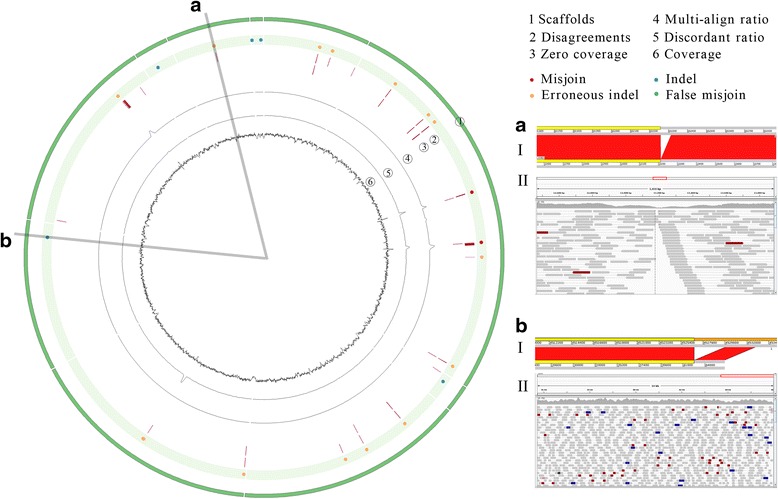
Visualization of misFinder output for identifying assembly errors on selected 10 scaffolds of most errors for *S.pombe* real data. The running results of misFinder are shown using Circos [30]. The ideogram (green) shows the circularized selected scaffolds. The scatter plot shows the marked assembly errors (red circles for misjoins, orange circles for indel errors) and correct assemblies (blue circles for correct indels, green circles for false misjoins) corresponding to structural variations identified by misFinder. The disagreement plot marks the disagreement for each base in scaffolds. The zero coverage plot marks each nucleotide with zero coverage. The multi-align ratio plot shows the ratio of multiple aligned reads for each region of 500 bp, ranging from 0 to 1. The discordant ratio plot shows the discordant ratio of discordant read pairs for each 500-bp region in scaffolds, ranging from 0 to 1. The last plot shows the read coverage in scaffolds. **a**, **b** show zoomed regions in the figure. **a** An identified assembly error of 55 bp deletion error in scaffolds was visualized using ACT (I) and IGV (II). **b** A detected correct assembly corresponding to structural variation of 3 kbp copy number variation in scaffolds was visualized using ACT (I) and IGV (II)

Figure 6 showed 10 scaffolds with the most typical errors and correct assemblies corresponding to variations were selected and analyzed by manually comparing the scaffolds with the reference sequence using ACT [27], and by visualizing their aligned paired-end reads using IGV [26]. In the figure, the erroneous regions had some disagreements, low coverage, many discordant read pairs, and other abnormal features, whereas the correct assembly regions had well supported paired-end reads, even coverage and no disagreements.

From the above three experiments, it shows that misFinder has good performance on identification of assembly errors with only few false positives, and it also pinpoints the correct assemblies corresponding to structural variations with high accuracy, which indicates the great power of misFinder to detect the assembly errors using genome reference and multiple features extracted from paired-end reads data, thus could help to increase the accuracy of assembly results for downstream analysis.

### Corrected assembly statistics

To highlight the performance of misFinder on mis-assembly identification, we evaluated it on both the accuracy of mis-assembly identification and the continuity of the corrected assembly using the assemblies of *E.coli* and *S.pombe* on simulated and real Illumina paired-end reads data. The *E.coli* and *S.pombe* assembly data were used again to perform the experiments, and the artificially modified reference was used as the high quality *E.coli* genome reference, and there were some differences between these two target genomes and their genome references.

In order to test the performance of misFinder on large genome, human chromosome 14 (refSeq: NT_026437.12, reference size 88.29 Mbp) simulated data were used to perform the experiments. The simulated data were generated using GemSIM [23], with length 100 bp, mean insert size 369 bp and standard deviation 45 bp. We chose CABOG [28] to perform the assembly because CABOG produced more errors than other tools according to our previous work [11, 12].

We compare the N50 size of the assemblies before and after the correction (indicated as N50_cor). Since QUAST [16] just computed mis-assembly statistics rather than correcting errors, we chose the metrics NA50 as the corrected N50. And we computed the following mis-assembly statistics, including true positives (TP, the assembly errors correctly determined), false positives (FP, correct assembly were incorrectly considered as assembly errors), false negatives (FN, assembly errors could not be determined), precision (*Precision* = *TP*/(*TP* + *FP*)) and true positive rate (TPR, *TPR* = *TP*/(*TP* + *FN*)). Precision is the fraction of identified assembly errors that are true, while true positive rate TPR (also known as recall or sensitivity) is the fraction of true assembly errors that are identified.

We also further compared misFinder with the reference-based approach QUAST (v2.3) [16] and the *de novo* approach REAPR (v1.0.17) [19] on the same datasets, and recorded their running time and memory consuming. The experiments for the *E.coli* simulated reads data and *S.pombe* real reads data were carried out on a 64-bit Linux machine with an Intel(R) Core-2 CPU 2.53-GHz supplied with 3 GB memory, and the results were shown in Tables 1 and 2, respectively. The experiments for the human chromosome 14 simulated reads data were carried out on a 64-bit Linux server with 64 Intel(R) Xeon(R) CPUs of 2.00-GHz supplied with 1 TB memory, and the results were shown in Table 3.

**Table 1:** Performance on assembly of *E.coli* simulated reads data

	#scf	N50 (kbp)	#scf_cor	N50_cor (kbp)	#Misass (TP,FP,FN)^a^	Precision	TPR	#Corr.^b^	Time (min)	Memory (GB)
misFinder	71	172.8	**74**	**172.8**	**27 / 0 / 1**	**1.0**	**0.964**	**8**	2	0.8
QUAST	71	172.8	-^c^	151.2	27 / 9 / 1	0.75	**0.964**	-	**1**	**0.4**
REAPR	71	172.8	**74**	**172.8**	13 / 0 / 15	**1.0**	0.464	-	17	**0.4**

^a^Number of Assembly errors were called by misFinder, QUAST and REAPR, including TP (true positives) and FP (false positives), while FN (false negatives) is the number of assembly errors that were not called

^b^Number of correct assemblies corresponding to structural variations called by misFinder

^c^QUAST did not output the number of broken scaffolds

The bold data reflected the best values for each column

**Table 2:** Performance on assembly of *S.pombe* real reads data

	#scf	N50 (kbp)	#scf_cor	N50_cor (kbp)	#Misass (TP,FP,FN)^a^	Precision	TPR	#Corr.^b^	Time (min)	Memory (GB)
misFinder	465	64.7	**481**	**61.6**	**113 / 3 / 9**	**0.974**	**0.926**	**22**	11	1.0
QUAST	465	64.7	-^c^	**61.6**	80 / 115 / 42	0.410	0.656	-	**2**	**0.8**
REAPR	465	64.7	668	40.1	59 / 891 / 63	0.062	0.484	-	128	**0.8**

^a^Number of Assembly errors were called by misFinder, QUAST and REAPR, including TP (true positives) and FP (false positives), while FN (false negatives) is the number of assembly errors that were not called

^b^Number of correct assemblies corresponding to structural variations called by misFinder

^c^QUAST did not output the number of broken scaffolds

The bold data reflected the best values for each column

**Table 3:** Performance on assembly of human chromosome 14 simulated reads data

	#scf	N50 (kbp)	#scf_cor	N50_cor (kbp)	#Misass (TP,FP,FN)^a^	Precision	TPR	#Corr.^b^	Time (min)	Memory (GB)
misFinder	2114	82.8	2552	69.9	**787 / 0 / 95**	**1.0**	**0.892**	**12**	**21**	30^c^
QUAST	2114	82.8	-^d^	69.4	602 / 282 / 265	0.681	0.694	-	107	**4**
REAPR	2114	82.8	**2322**	**75.3**	469 / 140 / 428	0.770	0.523	-	171	5.8

^a^Number of Assembly errors were called by misFinder, QUAST and REAPR, including TP (true positives) and FP (false positives), while FN (false negatives) is the number of assembly errors that were not called

^b^Number of correct assemblies corresponding to structural variations called by misFinder

^c^Memory usage of misFinder: Blastn 30 GB, error identification 4.5 GB

^d^QUAST did not output the number of broken scaffolds

The bold data reflected the best values for each column

#### Performance on assembly of *E.coli* simulated reads data

For the results in Table 1 on *E.coli*, misFinder identified 27 true positives in scaffolds, with 1 false negative and no false positives, the highest precision (100 %) and true positive rate (96.4 %). In the scaffolds, there were three large misjoins of distinct genome regions, misFinder correctly identified all of them and broke the scaffolds at their breakpoints. QUAST identified 36 mis-assemblies, however, there were 9 false positives and 1 false negative, and most of the false positives were corresponding to structural variations, so its precision was only 75 %. misFinder found 8 correct assemblies corresponding to structural variations using the artificially mutated genome reference, whereas QUAST also found these 8 correct assemblies, but it treated them as assembly errors. REAPR identified 13 errors without false positives, however, 15 assemblies errors were missing called, so the true positive rate TPR was only 46.4 %.

The corrected N50 size of misFinder and REAPR did not dropped (172.8 kbp), whereas the corrected N50 size of QUAST dropped from 172.8 kbp to 151.2 kbp, because it incorrectly broke the scaffolds at their breakpoint regions of differences due to structural variations. QUAST had the least time consumption (1 min), REAPR is the most time consuming (17 min), misFinder is moderate on time consumption (2 min), which is because that misFinder and REAPR performed the time consuming paired-end reads alignment while QUAST did not. QUAST and REAPR had the least memory usage (0.4 GB), and misFinder cost more memory (0.8 GB).

#### Performance on assembly of *S.pombe* real reads data

For the *S.pombe* assembly in Table 2, misFinder identified 116 errors including 113 true positives and 3 false positives (precision 97.4 %), with only 9 false negatives, and it also identified 22 correct assemblies corresponding to structural variations. In scaffolds, misFinder detected 22 misjoins and broke the scaffolds at their breakpoints. For QUAST, it identified 195 errors including 80 true positive errors and 115 false positives (precision 41 %), and with 42 false negatives. REAPR identified 950 errors including 59 true positives and 891 false positives (precision 6.2 %), and with 63 false negatives. misFinder detected more true positives than QUAST and REAPR, while QUAST and REAPR generated more false positives than misFinder.

misFinder had the highest precision (97.4 %), while these values of QUAST and REAPR were only 41 % and 6.2 %, respectively. QUAST and REAPR treated gaps between contigs in scaffolds as errors directly, while these gaps were caused by lack of read coverage and they were normal and common in scaffolds, so misFinder just report these gaps rather than treat them as errors. Moreover, QUAST treated all the differences as assembly errors directly without considering structural variations, thus introduced some false positives, e.g., there were 5 false positives caused by structural variations in the 115 false positives identified by QUAST, but misFinder could distinguish these correct assemblies corresponding to structural variations.

misFinder had the highest true positive rate (92.6 %), while these values of QUAST and REAPR were 65.6 % and 48.4 %, respectively. misFinder and QUAST employed the reference to anchor differences which may be assembly errors and generated accurate results, while QUAST had some missing calls in the scaffolds and introduced some false negatives. REAPR used the inconsistence information of read coverage which was not reliable on some error regions, thus resulted in some false negatives.

From the results, it can be seen that misFinder could give accurate assembly error calls, it is because that misFinder adopted a mixed approach to combine the high quality reference and paired-end reads information, and applied the genome reference information to locate the differences between the assembly and the reference, and then aligned the paired-end reads information to validate these differences according to multiple characteristics, such as disagreements, coverage, discordant reads and multiple aligned reads information.

misFinder and QUAST had slight decrease on N50 size which dropped from 64.7 kbp to 61.6 kbp, while for REAPR, the N50 size dropped dramatically from 64.7 kbp to 40.1 kbp, that was because REAPR broke scaffolds at assembly errors over gaps, and many gaps were caused by lack of read coverage which were normal and common in scaffolds, thus introduced false broken, whereas misFinder just broke scaffolds at regions of misjoins instead. QUAST was the fastest (2 min) and REAPR was the most time consuming (128 min), while misFinder was moderate on running time (11 min) and memory consumption (1.0 GB).

From the above experiments, misFinder had better performance than QUAST and REAPR on both simulated and real paired-end reads data. It generated more accurate assembly error calls because it combined the reference-based approach and *de novo* approach to fully utilize the information of high quality reference and paired-end reads in an unbiased way. It identified true positive mis-assemblies with few false positives and false negatives; it also distinguished the correct assemblies corresponding to structural variations from mis-assembled sequences.

#### Performance on assembly of human chromosome 14 simulated reads data

Human chromosome 14 simulated short reads data were used to test the performance of misFinder on large genomes. Six modifications were introduced to the human chromosome 14 (refSeq: NT_026437.12, reference size 88.29 Mbp), including one large relocation (segment size 70 kbp), one duplicated sequence (segment size 1.4 kbp), two insertions (70 bp and 30 bp) and two deletions (70 bp and 30 bp) (Additional file 1: Figure S9). We treated this mutated reference as new high quality reference, and treated the CABOG assembly as the target genome in which some differences may be due to SVs rather than assembly errors. As the duplicated sequence introduced one difference and the large relocation produced three differences at their joined positions, there were eight differences between the target genome and the reference. Moreover, we compared misFinder with QUAST and REAPR, and the results were shown in Table 3.

From the table, misFinder identified 787 true positives in scaffolds, with only 95 false negatives and no false positives, the highest precision (100 %) and true positive rate (89.2 %). QUAST identified 602 true positive mis-assemblies, however, there were 282 false positives and 265 false negatives, in which 6 false positives were corresponding to structural variations, so its precision and true positive rate were only 68.1 % and 69.4 %, respectively. misFinder found 12 correct assemblies in which 8 were corresponding to structural variations, whereas QUAST found only 6 of these 8 correct assemblies, but it treated them as assembly errors. REAPR identified 469 true positives with 140 false positives and 428 false negatives, so its precision and true positive rate were only 77 % and 52.3 %, respectively.

We checked the 4 false structural variations, and all these miscalls were caused by highly repetitive short tandem repeats with lengths larger than the read length (e.g., 100 bp), and one typical example was the deletion error of length 10 base pairs, which was caused by the highly repetitive short tandem repeat in the form of “CTTTCTTT…CTTTCCTTTCCTTT…CCTTT” with CTTT and CCTTT repeated many times, and reads in the genome region were well-aligned and without abnormal patterns because of the short size of the deleted sequence, so this case was difficult to be distinguished between the assembly error and structural variation. Therefore, misFinder identified these 8 structural variations correctly and miscalled other 4 assembly errors as structural variations.

misFinder and QUAST had decrease on N50 size which dropped from 82.8 kbp to 69 kbp, while for REAPR, the N50 size dropped slightly from 82.8 kbp to 75.3 kbp, that was because REAPR had much more false negatives than misFinder and QUAST, resulting in many assembly errors undetected.

misFinder had the least time consuming (21 min) and the highest memory consumption (30 GB), because it used 64 threads to perform the BLASTN alignment, and each thread required about 0.5 GB memory, thus it had the fastest speed and highest memory usage. If the thread number is half reduced to 32, the memory consumption will half reduced accordingly, and the running time may be doubled to about 40 min. Besides the BLASTN memory consumption, other parts of misFinder were low (4.5 GB). QUAST and REAPR took much more time (107 min and 171 min) and less memory usage (4 GB and 5.8 GB).

### Performance on close reference genomes with different similarities

In order to illustrate the impact of similarities on the performance between close genomes, four *E.coli* reference genomes with similarities ranging from 70.41 % to 99.56 % compared to *E.coli* K12 MG1655 (refSeq: NC_000913.2, genome size 4.64 Mbp), including *E.coli* O157:H7 str. Sakai (refSeq: NC_002695.1, genome size 5.5 Mbp, similarity 70.41 %), *E.coli* HS (refSeq: NC_009800.1, genome size 4.64 Mbp, similarity 83.56 %), and two close genome *E.coli* K12 DH10B (refSeq: NC_010473.1, genome size 4.69 Mbp, similarity 94.0 %) and *E.coli* K12 W3110 (refSeq: NC_007779.1, genome size 4.65 Mbp, similarity 99.56 %). The 50× *E.coli* K12 MG1655 simulated data and MaSuRCA assembly were used again to test their performances using above different reference genomes. As the main purpose of our work is to identify assembly errors to improve the assembly quality, we analyzed the identified errors and calculated the precision (*Precision* = *TP*/(*TP* + *FP*)) for the number of true errors among the identified errors for different reference genomes, and the results were shown in Table 4.

**Table 4:** Performance on close reference genomes with different similarities for the assembly of *E.coli* simulated reads data

Strain	Similarity compared to *E.coli* K12 MG1655	#Identified errors	#True errors	Precision
*E.coli* O157:H7 str. Sakai	0.7041	46	34	0.7391
*E.coli* HS	0.8356	38	32	0.8421
*E.coli* K12 DH10B	0.94	30	29	0.9667
*E.coli* K12 W3110	0.9956	30	30	1.0

From the table, it can be seen that the precision increased with the increase of the genome similarity. The *E.coli* strain Sakai had the lowest similarity (70.41 %) compared to the strain K12 MG1655 while the strain K12 W3110 had the highest similarity (99.56 %), and misFinder had the highest precision (100 %) on the strain K12 W3110 and the lowest precision (73.91 %) on the strain Sakai. misFinder identified more assembly errors on the strain Sakai than on other strains, however, some of the identified errors were false errors, they were miscalled because these scaffold regions were not well aligned to the reference, and some paired-end reads were also incorrectly aligned to these regions and caused some abnormal patterns (e.g., disagreements and abnormal coverage depth), even though these regions were correctly assembled and could be perfectly aligned to the *E.coli* K12 MG1655 reference.

For the reference genome of lower similarity, there will be more scaffold regions that could not be well aligned to the reference, and some regions may contain incorrectly aligned paired-end reads with some abnormal patterns even though these scaffold regions are correctly assembled, thus these regions may be error prone to be miscalled, whereas they may be ignored when using a higher similarity reference genome as they might be well aligned to the higher similarity reference. Therefore, higher similarity reference genome may lead to better results, and we recommend the similarity of the close reference as much higher as possible.

### Performance on identifying structural variations

One of the main features of misFinder is finding SVs between a close reference and an assembly, we tested misFinder on *E.coli* K12 MG1655 and *S.pombe* jb1168 genomes and compared its performance with Lumpy (v0.2.11) [29], a well-known SV finding tool, and the results were shown in Table 5. The *E.coli* MG1655 simulated reads data and the artificially modified reference, and the *S.pombe* jb1168 real reads data (SRA: ERX174934) and the close reference of *S.pombe* 972 h- were used again to perform the experiments.

**Table 5:** Performance on identifying structural variations for *E.coli* and *S.pombe* genomes

		misFinder			Lumpy		
Organism	Close reference genome	TP,FP,FN	Precision	TPR	TP,FP,FN	Precision	TPR
*E.coli* MG1655	*E.coli* MG1655 artificially mutated reference	8/0/0	1.0	1.0	4/1/4	0.8	0.5
*S.pombe* jb1168	*S.pombe* 972 h-	21/1/1	0.9545	0.9545	3/3/19	0.5	0.1364

We computed the following statistics, including true positives (TP, the SVs correctly determined), false positives (FP, false SVs were incorrectly considered as SVs), false negatives (FN, SVs could not be determined), precision (*Precision* = *TP*/(*TP* + *FP*)) and true positive rate (TPR, *TPR* = *TP*/(*TP* + *FN*)). Precision is the fraction of identified SVs that are true, while true positive rate TPR (also known as recall or sensitivity) is the fraction of true SVs that are identified. As the assembly of the mitochondrion of *S.pombe* (refSeq: NC_001326.1) were fragmented too much (scaffolds were typically 300–500 bp in length), it was difficult to determine whether the identified SVs were true or false, so the mitochondrion of *S.pombe* was excluded from the analysis.

According to the results, misFinder identified more structural variations with fewer false positives and fewer false negatives, and obtained better precision and higher true positive rate TPR on both of the two genomes than Lumpy on identifying structural variations. For the *E.coli* genome, there were 8 structural variations according to the artificially modifications (Additional file 1: Figure S8), misFinder identified all of them without introducing false positives and false negatives, whereas Lumpy discovered only 5 SVs with one false positive and 4 false negatives. For the *S.pombe* genome, misFinder identified 22 SVs with one false positive and one false negative, whereas Lumpy discovered only 6 SVs with 3 false positives and 19 false negatives, resulting the presision and TPR were only 0.5 and 0.14, respectively.

Moreover, there were some novel sequences in *S.pombe* jb1168 compared to *S.pombe* 972 h-, misFinder identified these novel sequences as structural variations while Lumpy could not find these novel sequences because Lumpy does not contain the assembly process. For example, there were 5 novel sequences in *S.pombe* jb1168 with lengths ranging from 1 kbp to 9 kbp compared to *S.pombe* 972 h-, but Lumpy could not find these novel sequences.

## Discussion

We have developed an open-source mis-assembly identification method, misFinder, which identifies the assembly errors by combining the reference and paired-end reads information. The main purpose of our work is to improve the assembly quality by identifying the mis-assemblies excluding the differences caused by structural variations between the target genome and the reference genome.

There are many repeats (or duplicated sequences) in genome, and they are difficult to be resolved in assembly. Therefore, assembly is usually broken in these repeat regions, and as a result, the repetitive sequences appear at the scaffold ends in most cases, only a few repeats occur in the inner parts of scaffolds. This is consistent with our experiments.

For the repeats, they should be considered together with the scaffolds they belonged to, even though they have multiple aligned locations in the reference. So, if the repeats are well aligned to the reference together with their scaffolds, the other locations of the repeats will not be considered in our method. Therefore, most of the duplicated sequences have no much impact to the mis-assembly identifications.

For the structural variation identification, paired-end reads are aligned to the reference (not the scaffolds, and in fact, assembly is usually not performed in most cases), and some abnormal patterns of paired-end reads are applied to call structural variations. However, in our method, scaffolds are first aligned to the reference to determine their differences, and these differences may be caused either by mis-assemblies or by structural variations. And then, paired-end reads are aligned to the scaffolds (not the reference) to distinguish mis-assemblies using those abnormal patterns of paired-end reads. And according to our experiments, the differences that are due to structural variations usually have no abnormal patterns, thus can be excluded. Note that we only consider the scaffold regions that have differences with the reference rather than other well aligned scaffold regions.

However, some assembly errors caused by highly repetitive short tandem repeats are difficult to be correctly resolved, some of them may be miscalled as structural variations because the lengths of inserted/deleted sequences are typically short (10–20 bp) and their breakpoint regions usually have well-aligned paired-end reads and no abnormal patterns. Some of these short tandem repeats are difficult to be resolved by misFinder and may cause some mis-identifications, and the lengths of these highly repetitive short tandem repeats are usually longer than the read length, and we think that increase the read length may help to identify these assembly errors more accurately.

Moreover, some scaffold regions have some mismatches and abnormal coverage depth even though these regions are perfectly aligned to the reference. The reason is that these regions are similar with some other genomic regions which are not successfully reconstructed during assembly (we call these regions as missing regions), and paired-end reads derived from these missing regions are incorrectly aligned to the similar regions with some mismatches, and as a result, abnormal patterns are shown in some well aligned scaffold regions (Additional file 1: Figure S10). But, these regions are well aligned to the reference (they have no differences), so they are correctly constructed, and we do not consider these well aligned regions to prevent miscalls in our method.

## Conclusions

Even though there is a high-quality genome reference, for genome sequencing (even the same species), there are usually some differences between the target genome and the reference genome as the two genomes are not exactly the same. For the assembly of a target genome, it may contain many differences may be caused by assembly errors or structural variations. When calling the assembly errors, if we do not consider the differences between the target genome and the reference genome, the results may contain some biases.

In this article, we present misFinder, a tool that aims to identify the assembly errors with high accuracy in an unbiased way and correct these errors at their misjoined positions to improve the assembly accuracy before downstream analysis. It uses the reference (or close related reference) to find the differences between the scaffolds and the reference, and uses multiple features extracted from the paired-end reads to validate these differences to determine whether they are assembly errors or correct assemblies corresponding to structural variations. Experiments showed that misFinder could identify the assembly errors with fewer miscalls, and the correction almost has no much impacts on the continuity of the assembly both for simulated *E.coli* reads data and real *S.pombe* dataset. Human chromosome 14 experiments showed that misFinder could identify the assembly errors and correct them to improve the assembly quality for large genomes.

## Availability of supporting data

misFinder was implemented in C language on Linux x86_64 machine. The source code can be freely downloaded from https://github.com/hitbio/misFinder.

## Additional file

Additional file 1:
**Pattern screenshots for assembly errors.** The file gives screenshots of different patterns for assembly errors, including misjoins, insertions and deletions for the assembly on *E.coli* simulated paired-end short reads data. The file also gives the screenshots of identified novel sequences for the *S.pombe* strain jb1168 genome comparing to the reference of *S.pombe* strain 972 h-. The file still gives some detailed information for the artificially modified references for *E.coli* and human chromosome 14. (DOC 2626 kb)
