# Supplementary material for: misFinder: identify mis-assemblies in an unbiased manner using reference and paired-end reads
Source: BMC Bioinformatics. 2015 Nov 16;16:386. doi: 10.1186/s12859-015-0818-3 (PMC4647709; doi:10.1186/s12859-015-0818-3)
Supplement: Additional file 1: — Pattern screenshots for assembly errors. The file gives screenshots of different patterns for assembly errors, including misjoins, insertions and deletions for the assembly on E.coli simulated paired-end short reads data. The file also gives the screenshots of identified novel sequences for the S.pombe strain jb1168 genome comparing to the reference of S.pombe strain 972 h-. The file still gives some detailed information for the artificially modified references for E.coli and human chromosome 14. (DOC 2626 kb) [file 12859_2015_818_MOESM1_ESM.doc]

Additional file

# misFinder: Identify mis-assemblies in an unbiased manner using reference and paired-end reads

Xiao Zhu^1,2^, Henry C.M. Leung^3^, Rongjie Wang^2^, Francis Y.L. Chin^3^, Siu Ming Yiu^3^, Guangri Quan^2^, Yajie Li^4^, Rui Zhang^4^, Qinghua Jiang^5^, Bo Liu^2^, Yucui Dong^6^, Guohui Zhou^1^, Yadong Wang^2§^

^1^College of Computer Sciences and Information Engineering, Harbin Normal University, Harbin, Heilongjiang, China

^2^Center for Bioinformatics, School of Computer Sciences and Technology, Harbin Institute of Technology, Harbin, Heilongjiang, China

^3^Department of Computer Science, University of Hong Kong, Pokfulam Road, Hong Kong

^4^The Fourth Affiliated Hospital of Harbin Medical University, Harbin, Heilongjiang, China

^5^School of Life Science and Technology, Harbin Institute of Technology, Harbin, Heilongjiang, China

^6^Department of Immunology, Harbin Medical University, Harbin, Heilongjiang, China

^§^Corresponding author

Correspondence should be addressed to: Yadong Wang

Email: [ydwang@hit.edu.cn](mailto:ydwang@hit.edu.cn)

# 1 Features of assembly errors

The assembly was carried out on 50x *E.coli* (reference size 4.64 Mbp) simulated dataset using MaSuRCA . We take it as an example to illustrate the different features of assembly errors (misjoin, insertion error and deletion error ).

## 1.1 Normal regions

For normal regions, they usually have no disagreements and concordant read pairs (Figure S1), which can be used as a comparison with the breakpoint regions of mis-assemblies.


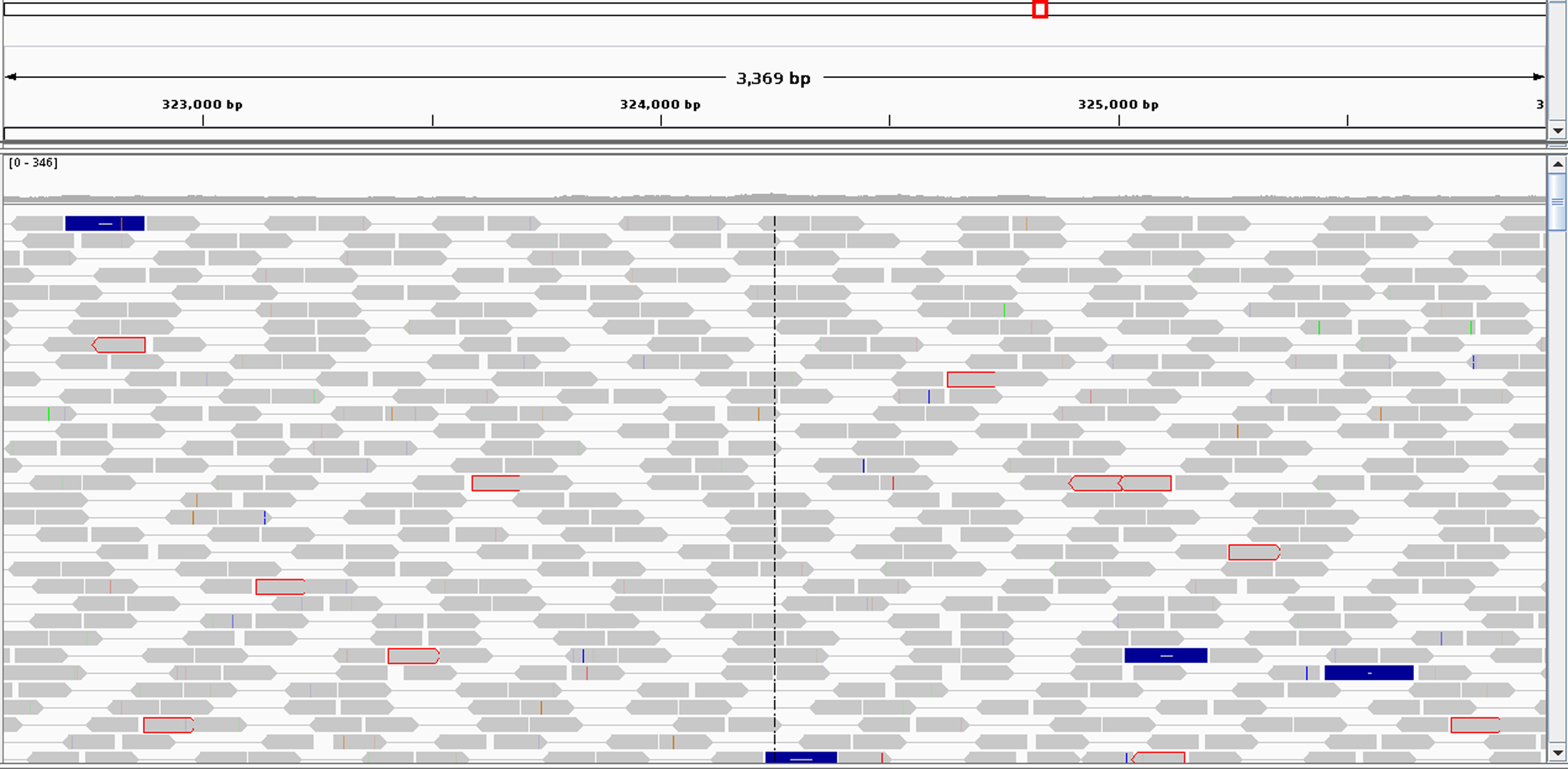


**Figure S1. Normal region of a scaffold visualized by IGV** . The normal regions usually have even coverage, concordant read pairs and no disagreements.

## 2.2 Misjoins

There were two typical large misjoin assembly errors illustrated in Figures S2-S3, and the errors are caused by repeats with lengths larger than the insert size (mean size 368 bp, standard deviation 61.3 bp) of the paired-end library.

For these two misjoins, the coverage depth is high, and there are some disagreements and many multiple aligned reads, which are much different from the normal regions, thus these errors can be easily identified according to their features.


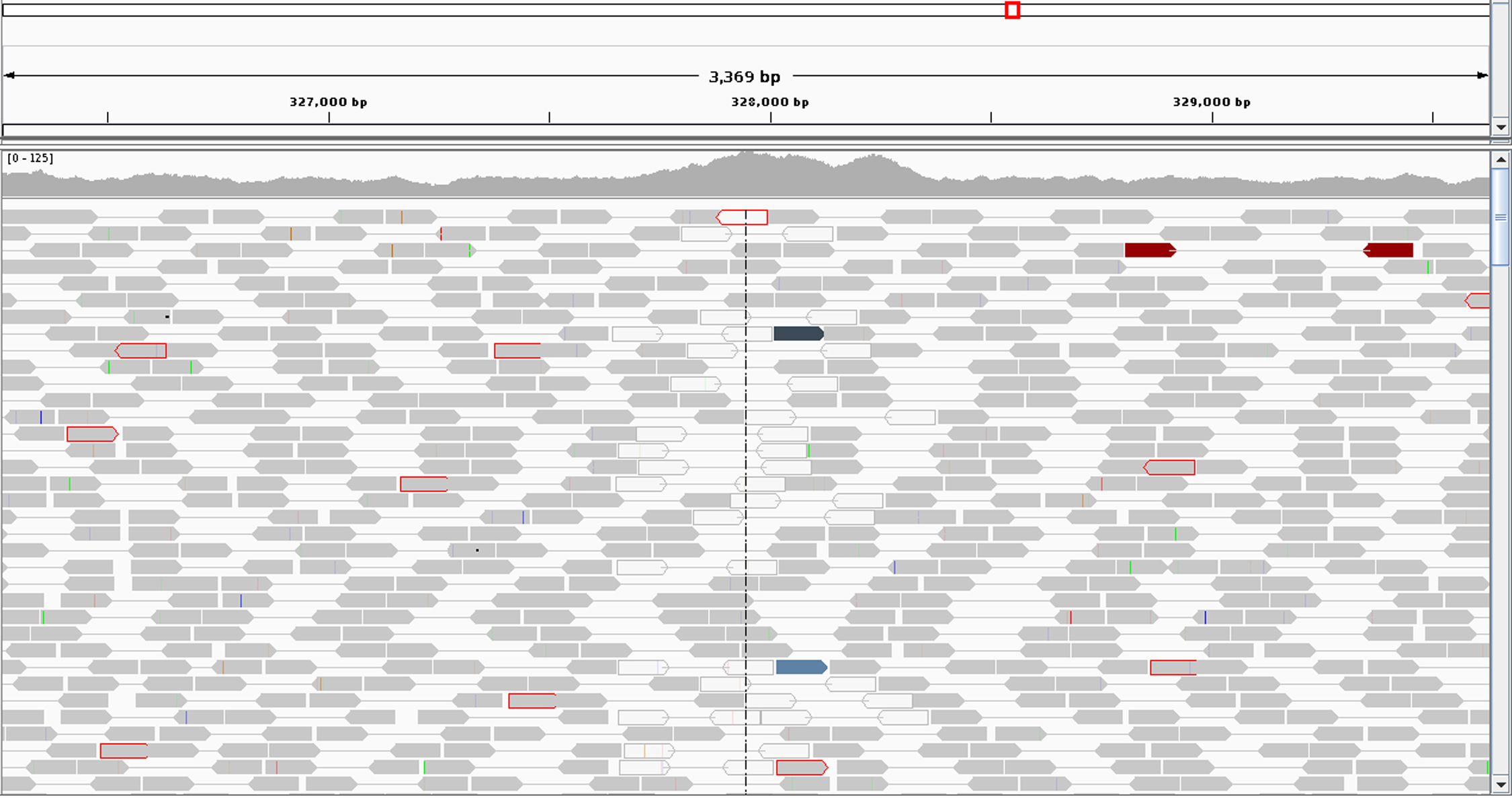


**Figure S2. A misjoin visualized by IGV**. It is caused by a repeat from scaffold region 327,620 - 328,385 which has high coverage, many multiple aligned reads (white rectangles with arrows), around the repeat margins.


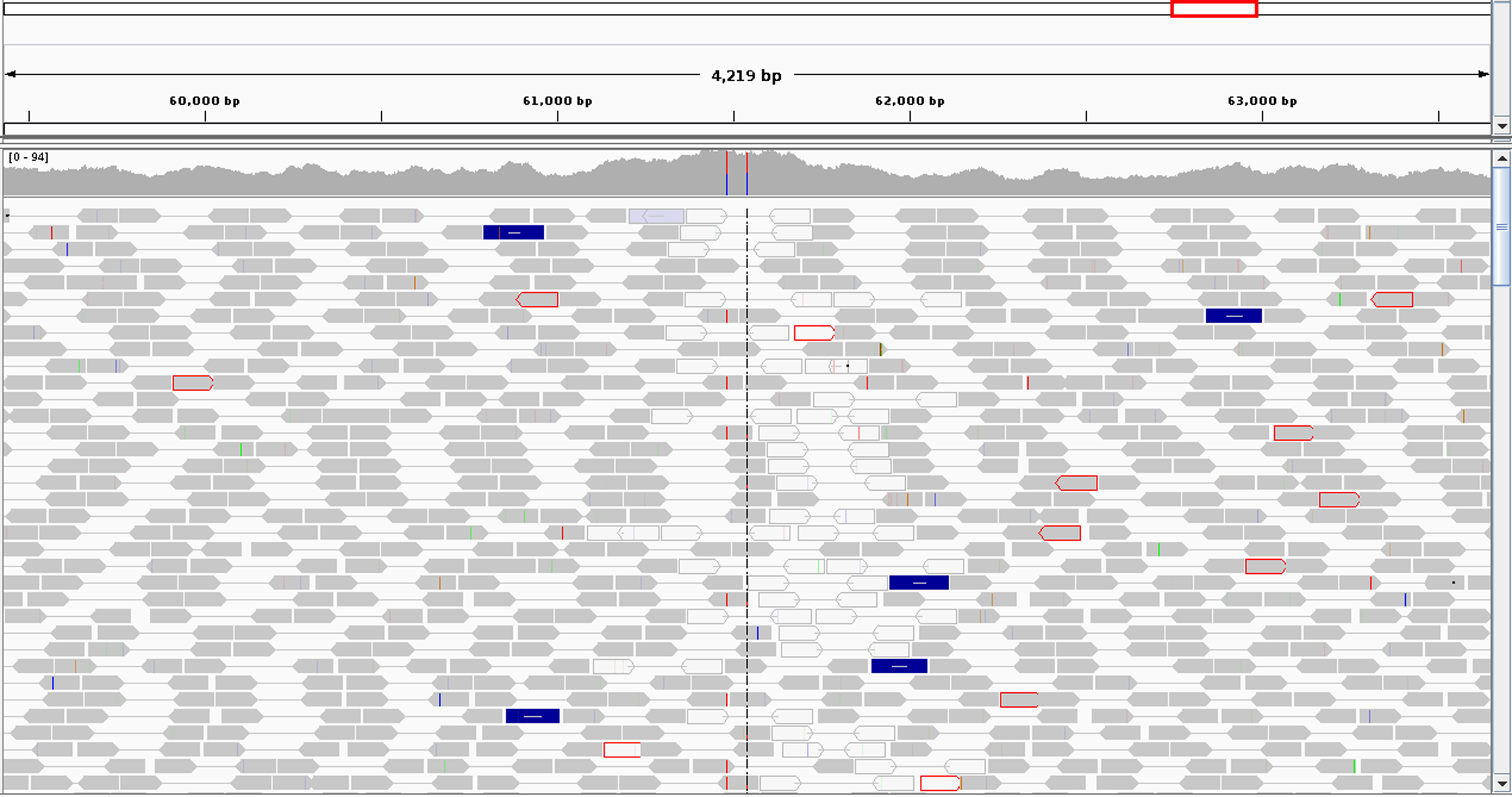


**Figure S3. Another misjoin visualized by IGV**. It is also caused by a repeat from scaffold region 61,085 - 62,217 which has many disagreements (color lines in IGV coverage panel), many multiple aligned reads (white rectangles with arrows), around the repeat margins.

## 3.3 Insertion errors and deletion errors

For an insertion error in scaffold in Figure S4, the fragment size of the paired-end reads around the repeat is much larger than the library insert size. Moreover, there are some disagreements and low read coverage depth in the error region.

For a deletion error in scaffold in Figure S5, the paired-end reads have a short insert size, there are some disagreements and abnormal read coverage depth in this region.


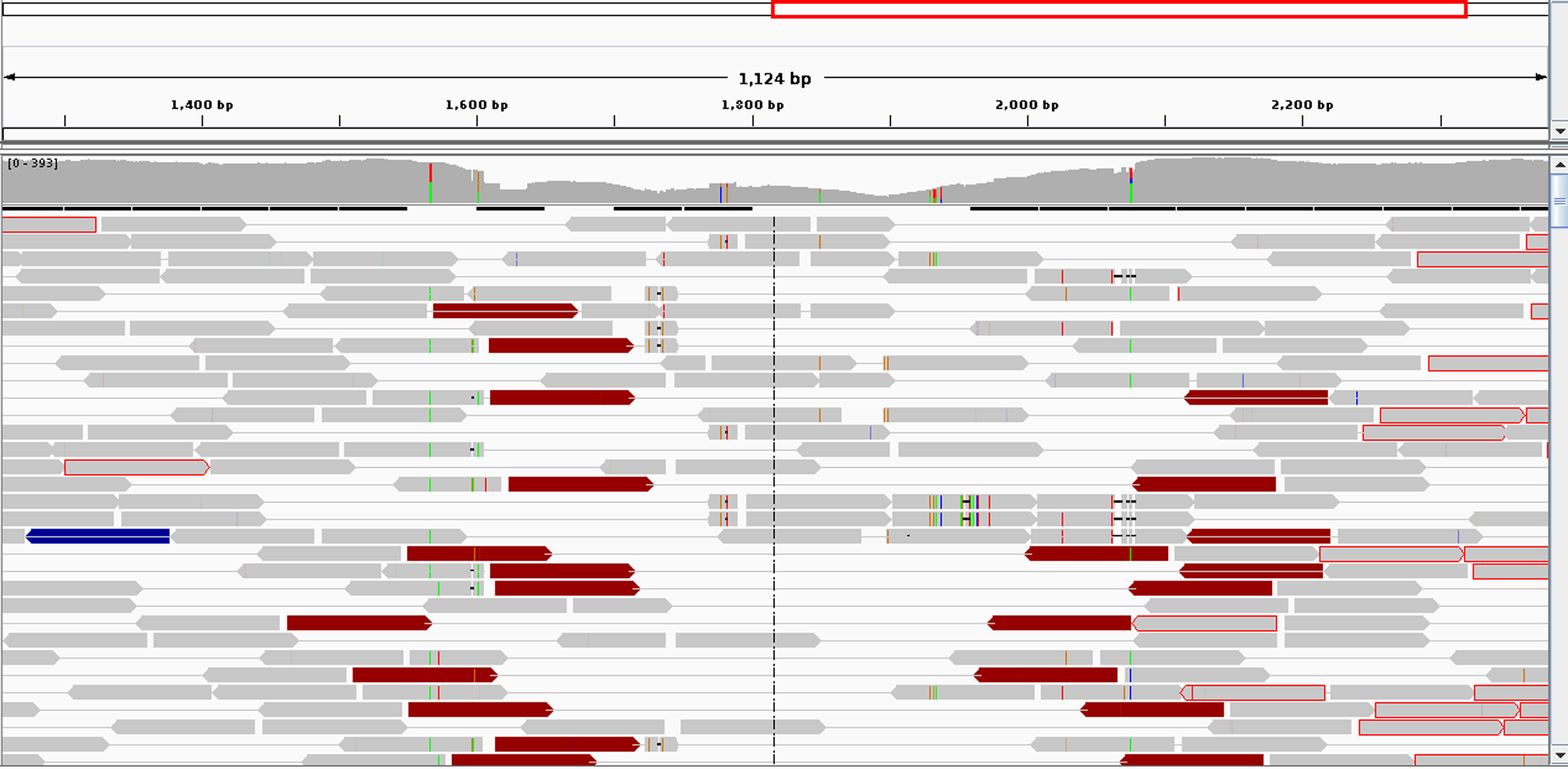


**Figure S4. An insertion error visualized by IGV.** As there was an inserted sequence of length 192 bp in the scaffold, it also had some disagreements around the breakpoint region 1737 - 1900, and the paired-end reads had a large fragment size and low coverage.


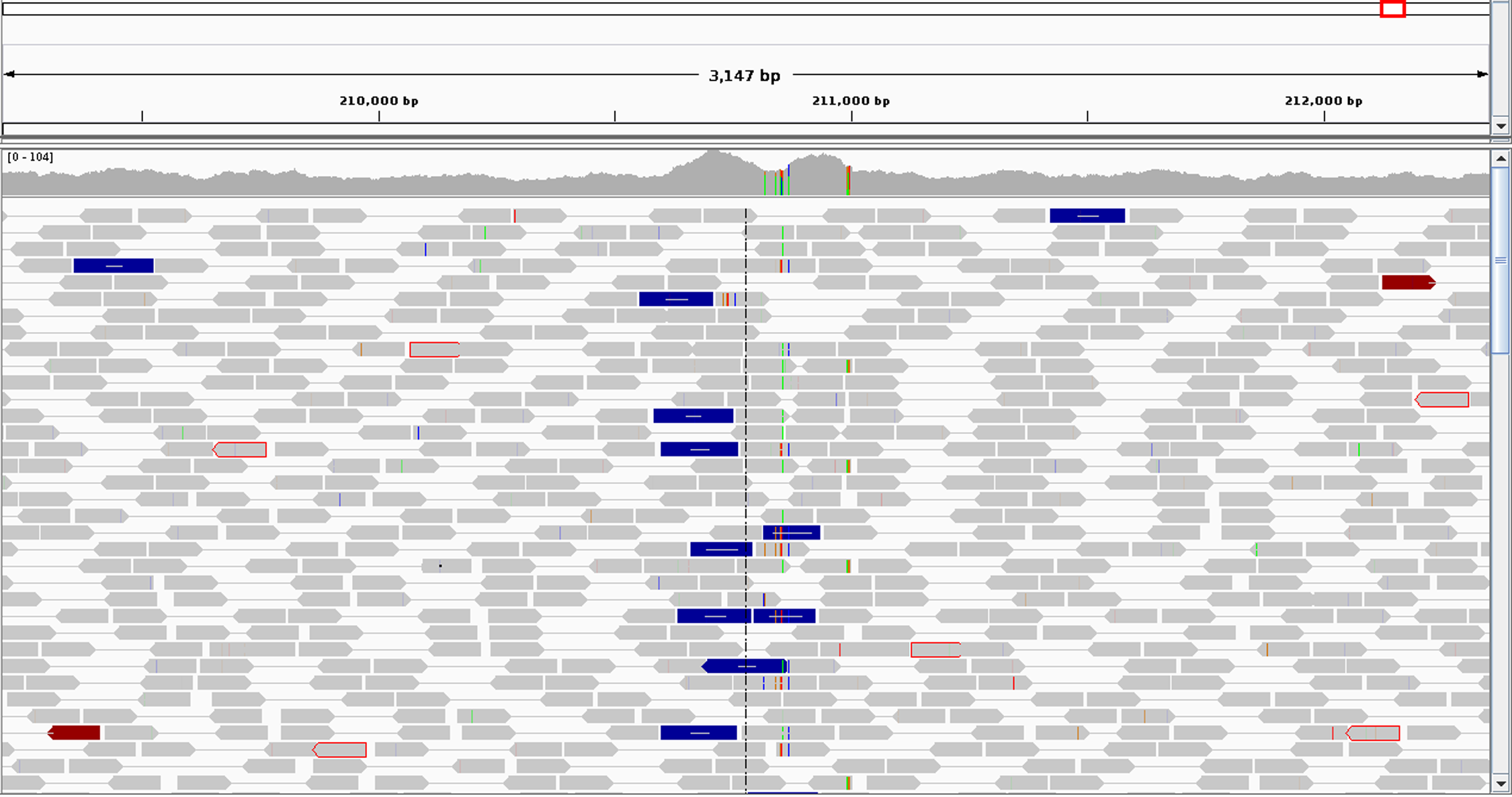


**Figure S5. A deletion error visualized by IGV.** As there was a deleted sequence of length 225 bp in the scaffold, it also had some disagreements, and the paired-end reads had a short fragment size.

# 2 Typical novel sequences in *S.pombe* jb1168 genome

We selected two large typical novel sequences in *S.pombe* strain jb1168 genome identified by misFinder compared to the *S.pombe* strain 972h- genome reference (Figures S6-S7). These two novel sequences had large unaligned segments of lengths 9 kbp and 4.6 kbp, respectively.


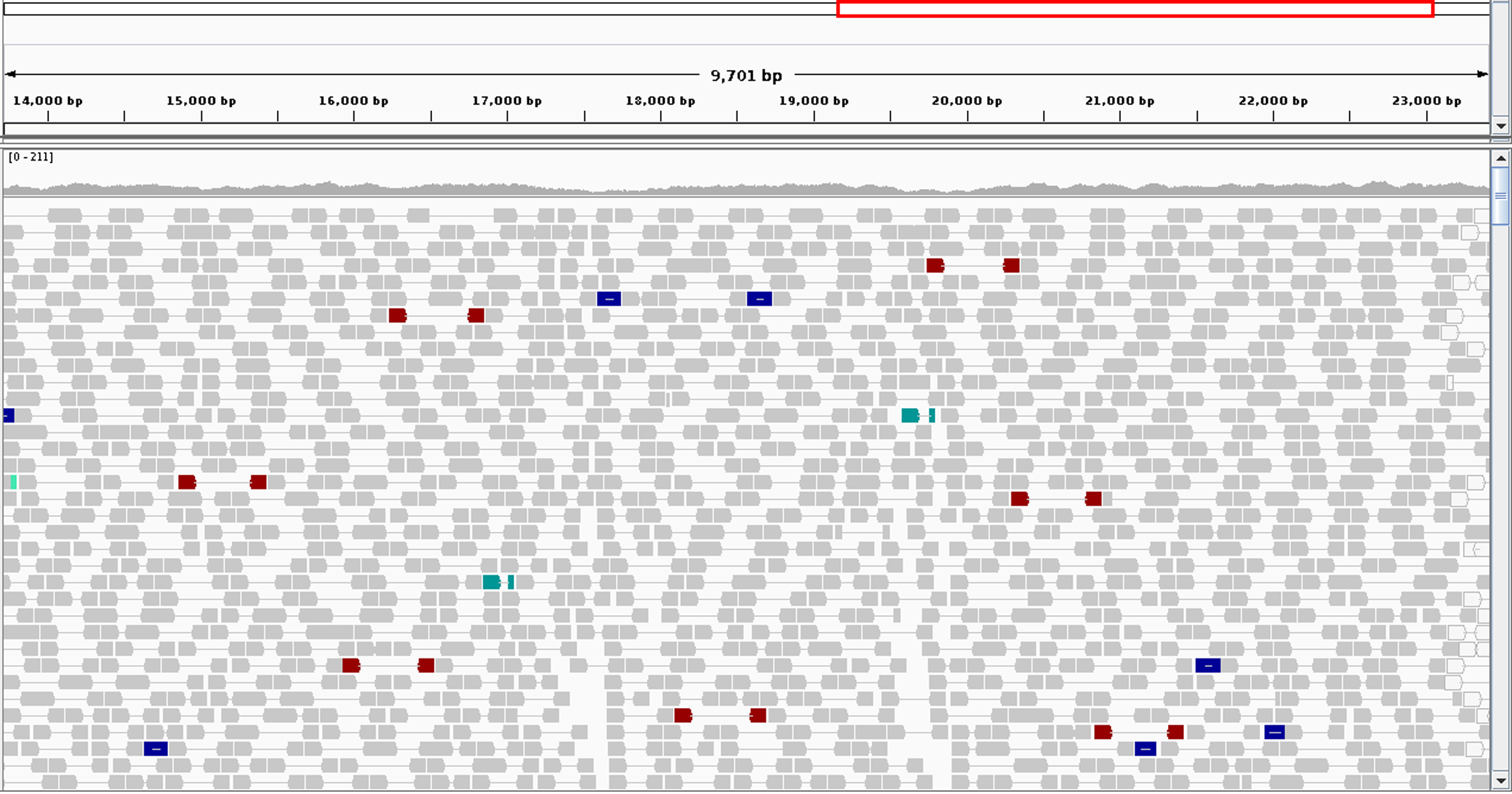


**Figure S6. Novel sequence of 9 kbp in *S.pombe* strain jb1168 genome compared to *S.pombe* strain 972h-.** The novel sequence of 9 kbp with scaffold positions from 14.1 kbp to 23.1 kbp was identified as correct assembly due to structural variation. The paired-end reads information was normal in this region.


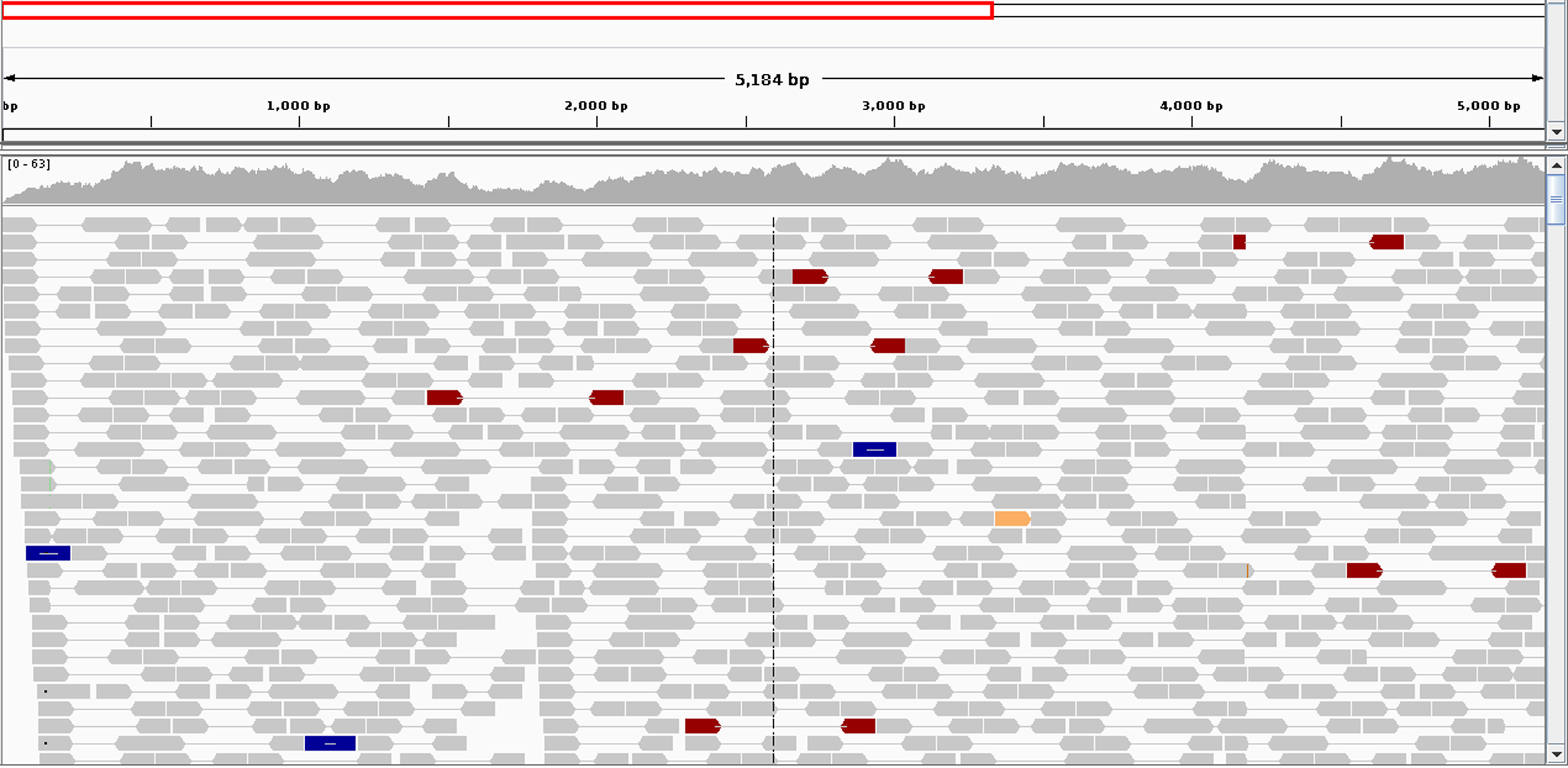


**Figure S7. Novel sequence of 4.6 kbp in *S.pombe* strain jb1168 genome compared to *S.pombe* strain 972h-.** The novel sequence of 4.6 kbp at the beginning of the scaffold was identified as correct assembly due to structural variation. The paired-end reads information was normal in this region.

# 3 Artificial modifications of *E.coli* reference

We introduced six different modifications into the *E.coli* MG1655 genome reference (refSeq: NC_000913.2) to analog the structural variations (SVs), these modifications included one duplicated sequence (segment size 1 kbp), one large relocation (segment size 57 kbp), two insertions (70 bp and 30 bp) and two deletions (70 bp and 30 bp) (Figure S8). The similarity between the modified reference and the original reference is 99.97%. We treated the mutated reference as the new reference, and the assembly as the target genome which contained SVs. As the large relocation produced three differences at their joined positions, there were eight differences between the target genome and the reference. As a result, misFinder identified the 27 assembly errors and determined all the 8 differences caused by structural variations as correct assemblies.


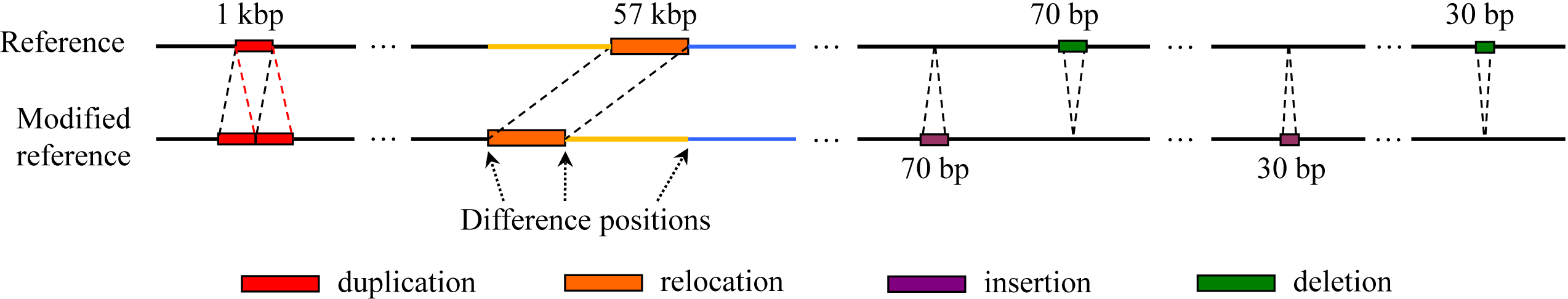


**Figure S8. Artificial modifications introduced into *E.coli* MG1655 reference.** These modifications included one duplication of 1 kbp, one large relocation of 57 kbp, two inserted sequences (70 bp and 30 bp) and two deleted sequences (70 bp and 30 bp). The relocation produced three differences at the joined positions.

# 4 Artificial modifications of human chromosome 14 reference

We introduced six different modifications into the human chromosome 14 reference (refSeq: NC_026437.12) to analog the structural variations (SVs), these modifications included one large relocation (segment size 70 kbp), one duplicated sequence (segment size 1.4 kbp), two insertions (70 bp and 30 bp) and two deletions (70 bp and 30 bp) (Figure S9). We treated the mutated reference as the new reference, and the assembly as the target genome which contained SVs. As the large relocation produced three differences at their joined positions, there were eight differences between the target genome and the reference. As a result, misFinder could successfully identify the 8 structural variations. However, there were 4 assembly errors were miscalled as structural variations, these miscalls were caused by short tandem repeats with lengths larger than the read length (e.g. 100 bp), one typical example was the deletion error of length 10 base pairs, which was caused by the short tandem repeat in the form of "CTTTCTTT…CTTTCCTTTCCTTT…CCTTT" with CTTT and CCTTT repeated many times, and reads in these genome regions were well aligned and without abnormal patterns because of the short size of the deleted sequence, so this case was difficult to be distinguished between the assembly error and structural variation. Therefore, misFinder identified these 8 structural variations correctly and miscalled other 4 assembly errors as structural variations.


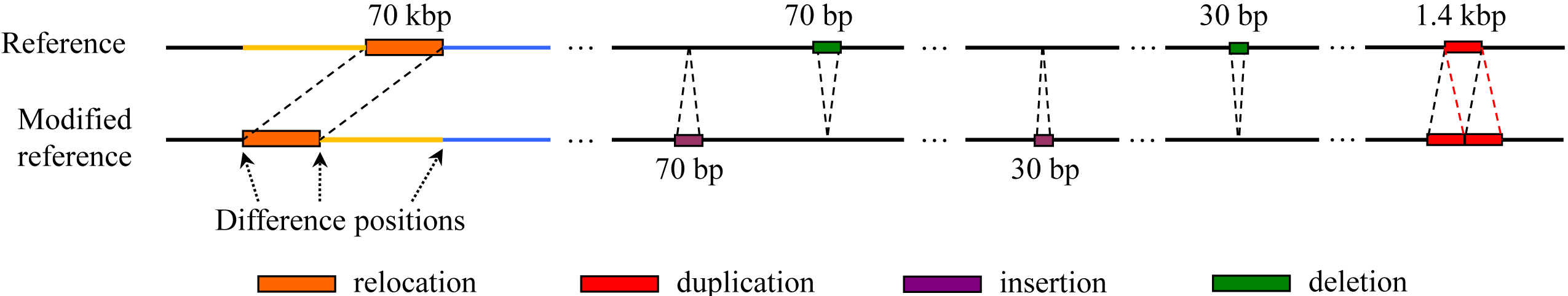


**Figure S9. Artificial modifications introduced into human chromosome 14 reference.** These modifications included one large relocation of 70 kbp, one duplication of 1.4 kbp, two inserted sequences (70 bp and 30 bp) and two deleted sequences (70 bp and 30 bp). The relocation produced three differences at the joined positions.

# 5 Abnormal patterns in some normal scaffold regions of *E.coli* assembly

After analyzing the scaffold regions of *E.coli* assembly, it is observed that some scaffold regions have some mismatches and abnormal read coverage depth even though these regions are perfectly aligned to the reference. The reason is that these regions are similar with some other genomic regions which are not successfully reconstructed during assembly (we call these regions as missing regions), and paired-end reads derived from these missing regions are incorrectly aligned to the similar regions with some mismatches, and as a result, abnormal patterns are shown in some well aligned scaffold regions (Figure S10). Therefore, we do not consider these well aligned regions to prevent miscalls in our method since they are well aligned to the reference.


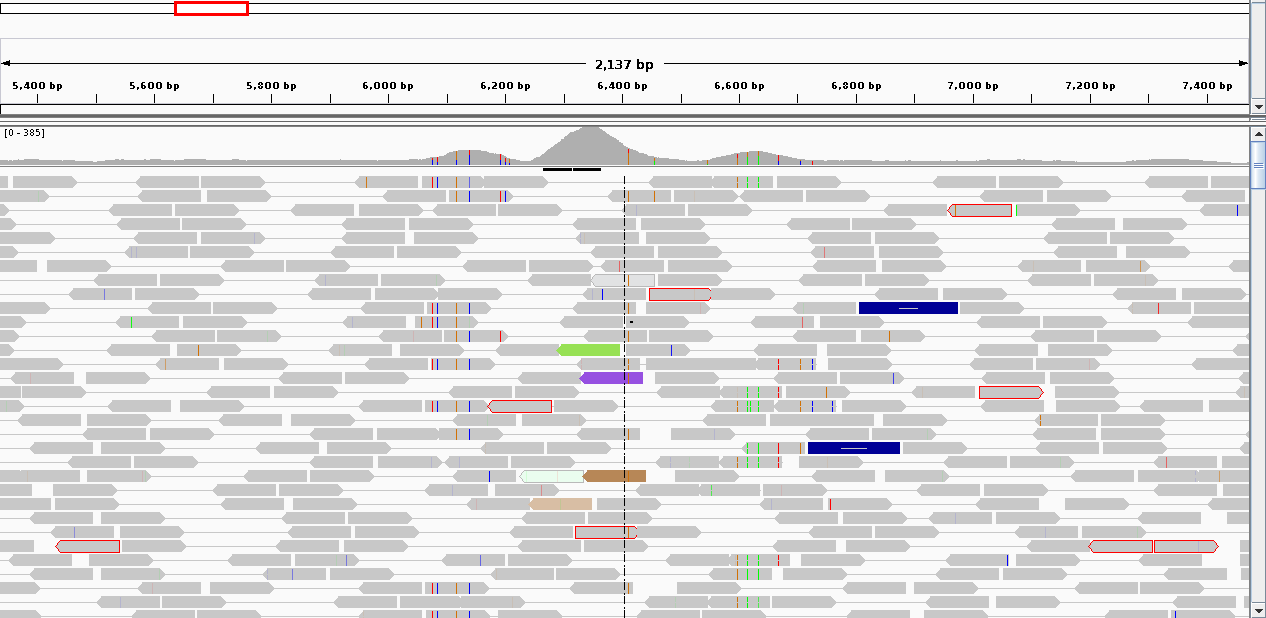


**Figure S10. Abnormal patterns in perfectly aligned correct scaffold region in *E.coli* assembly.** Abnormal coverage depth and many mismatches occurred in the well aligned region around 6.4 kbp of the scaffold.

# References

1. Zimin AV, Marcais G, Puiu D, Roberts M, Salzberg SL, Yorke JA: The MaSuRCA genome assembler. *Bioinformatics* 2013, 29(14):2669-2677.

2. Salzberg SL, Phillippy AM, Zimin A, Puiu D, Magoc T, Koren S, Treangen TJ, Schatz MC, Delcher AL, Roberts M *et al*: GAGE: A critical evaluation of genome assemblies and assembly algorithms. *Genome Res* 2012, 22(3):557-567.

3. Thorvaldsdottir H, Robinson JT, Mesirov JP: Integrative Genomics Viewer (IGV): high-performance genomics data visualization and exploration. *Briefings in Bioinformatics* 2013, 14(2):178-192.
